# Hemoglobin-mediated biomimetic synthesis of paramagnetic O_2_-evolving theranostic nanoprobes for MR imaging-guided enhanced photodynamic therapy of tumor

**DOI:** 10.7150/thno.46228

**Published:** 2020-09-19

**Authors:** Xiudong Shi, Weitao Yang, Qiong Ma, Yang Lu, Yan Xu, Kexin Bian, Fengjun Liu, Chunzi Shi, Han Wang, Yuxin Shi, Bingbo Zhang

**Affiliations:** 1 Department of Radiology, Shanghai Public Health Clinical Center, Fudan University, Shanghai 201508, China.; 2 Department of Radiology, Shanghai General Hospital, Shanghai Jiao Tong University School of Medicine, Shanghai 200080, China.; 3 The Institute for Translational Nanomedicine, Shanghai East Hospital; The Institute for Biomedical Engineering & Nano Science, Tongji University School of Medicine, Shanghai 200092, China.; 4 Translational Medicine Center, Shanghai Key Laboratory of Molecular Imaging, Shanghai University of Medicine and Health Sciences, Shanghai 201318, China.

**Keywords:** biomimetic synthesis, MR imaging, tumor hypoxia, photodynamic therapy, endogenous protein

## Abstract

The hypoxic microenvironment in solid tumors severely limits the efficacy of photodynamic therapy (PDT). Therefore, the development of nanocarriers co-loaded with photosensitizers and oxygen, together with imaging guidance ability, is of great significance in cancer therapy. However, previously reported synthetic methods for these multi-functional probes are complicated, and the raw materials used are toxic.

**Methods:** Herein, the human endogenous protein, hemoglobin (Hb), was used for the simultaneous biomimetic synthesis of Gd-based nanostructures and co-loading of Chlorine e6 (Ce6) and oxygen for alleviating the hypoxic environment of tumors and accomplishing magnetic resonance imaging (MRI)-guided enhanced PDT. The Gd@Hb^Ce6-PEG^ nanoprobes were synthesized *via* a green and protein biomimetic approach. The physicochemical properties, including relaxivity, oxygen-carrying/release capability, and PDT efficacy of Gd@Hb^Ce6-PEG^, were measured *in vitro* and *in vivo* on tumor-bearing mice after intravenous injection. Morphologic and functional MRI were carried out to evaluate the efficacy of PDT.

**Results:** The results demonstrated the successful synthesis of compact Gd@Hb^Ce6-PEG^ nanostructures with desired multi-functionalities. Following treatment with the nanoparticles, the embedded MR moiety was effective in lighting tumor lesions and guiding therapy. The oxygen-carrying capability of Hb after biomimetic synthesis was confirmed by spectroscopic analysis and oxygen detector *in vitro*. Further, tumor oxygenation for alleviating tumor hypoxia *in vivo* after intravenous injection of Gd@Hb^Ce6-PEG^ was verified by photoacoustic imaging and immunofluorescence staining. The potent treatment efficacy of PDT on early-stage was observed by the morphologic and functional MR imaging. Importantly, rapid renal clearance of the particles was observed after treatment.

**Conclusion:** In this study, by using a human endogenous protein, we demonstrated the biomimetic synthesis of multi-functional nanoprobes for simultaneous tumor oxygenation and imaging-guided enhanced PDT. The therapeutic efficacy could be quantitatively confirmed at 6 h post PDT with diffusion-weighted imaging (DWI).

## Introduction

Photodynamic therapy (PDT) entails energy transfer from an injected photosensitizer to molecular oxygen under light irradiation generating reactive oxygen species to eradicate tumor cells 1-3. Compared to traditional cancer treatments, PDT is an effective and promising treatment with the advantages of simple operation, high selectivity, and minimal invasiveness 4, 5. However, PDT is highly dependent on the local oxygen concentration of tumor lesions, and the hypoxic microenvironment in solid tumors limits the effectiveness of PDT treatment 6, 7. Therefore, delivering photosensitizers together with oxygen into tumor tissues is highly desired 8.

At present, a few approaches reported the co-delivery of photosensitizers and oxygen by using nanostructures, including oxygen-adsorbing porous nanomaterials like silica and metal-organic frame (MOF) and oxygen-carrying materials like perfluorohexane 9-12. PDT efficacy can also be enhanced either by *in-situ* production of oxygen in tumor tissues *via* chemical reactions or by circumventing the use of oxygen during PDT 13-17. Regardless of the efficiency, each strategy involves the fabrication of integrated multi-functional nanocomposites critical for clinical application.

Currently, protein-mediated biomimetic synthesis is an eco-friendly technology for fabricating nanomaterials with favorable green synthesis features, flexibility of functional integration and high biocompatibility 18-20. Biomimetic synthesis is generally regarded as a fascinating research area with great potentials in the biomedical fields. In this process, proteins play multiple roles, including reducing as well as stabilizing agents and reaction region providers 21. We previously used bovine serum albumin (BSA) to synthesize a series of nanoprobes, including single-function probes like gadolinium-based hybrid nanoparticles for angiography 22, and dual/multi-function probes like near infrared paramagnetic Ag_2_S quantum dots, and Gd:CuS@BSA nanoparticles for cancer imaging and photothermal therapy 20. Besides BSA, we also explored a human endogenous protein, glutathione S-transferase (GST), to synthesize a bimodal probe for cancer computerized tomography (CT)/magnetic resonance (MR) imaging 23. Our previous work and other published studies have shown advantages of proteins for the biomimetic synthesis of nanostructures, particularly for facile integration of multiple functionalities 20, 24-27.

In this study, we used the oxygen-carrier protein, hemoglobin amply produced in humans, and demonstrated retention of its oxygen-carrying ability following protein-mediated biomimetic synthesis of the paramagnetic nanoprobe with PDT function (Gd@Hb^Ce6-PEG^). Hemoglobin was chosen because of its biocompatibility as there are safety concerns with the chemosynthetic oxygen carriers. The second important reason was the intrinsic oxygen-carrying capability of hemoglobin in blood circulation 28, 29. Finally, the hemoglobin structure was conducive for the biomimetic synthesis of imaging nanoparticles and simultaneous loading photosensitizers for imaging-guided enhanced PDT. Our study demonstrated that besides serving as a stabilizing agent, providing reaction region in the biomimetic chemistry of Gd nanoparticles, and loading Ce6 molecules *via* hydrophobic binding force, hemoglobin maintains oxygen-carrying capability. The main physicochemical properties, including the size, morphology, relaxivity, oxygen-carrying/release capability, and PDT efficacy of Gd@Hb^Ce6-PEG^, were measured and confirmed *in vitro* and *in vivo* in tumor-bearing mice after intravenous injection. The MR moiety showed significant tumor uptake of Gd@Hb^Ce6-PEG^ with a wide time window for imaging, providing noninvasive precise guidance for PDT. The enhanced PDT treatment efficacy of oxygenated Gd@Hb^Ce6-PEG^ (oxy-Gd@Hb^Ce6-PEG^) was evaluated by functional MR imaging besides the routine morphologic MR imaging.

## Results and Discussion

### Synthesis and characterization of Gd@Hb^Ce6-PEG^ nanoparticles

**Scheme 1:**
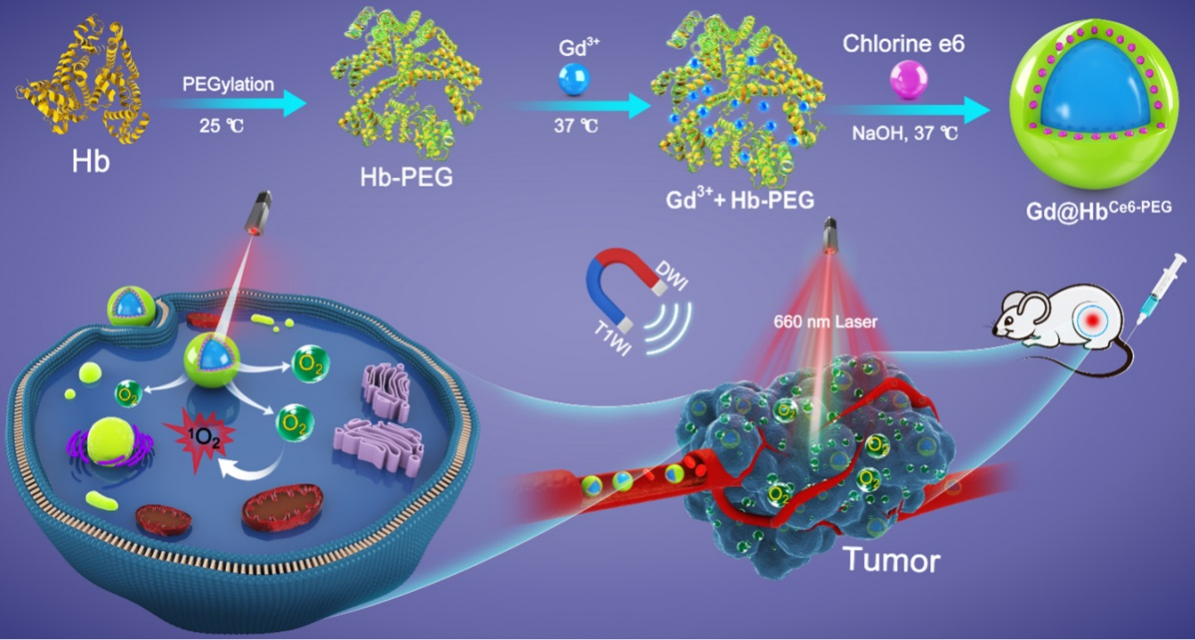
Schematic representation of the hemoglobin-mediated biomimetic synthesis of multi-functional nanoparticles for tumor oxygenation and MR imaging-guided enhanced PDT of tumors.

As depicted in Scheme 1, hemoglobin was first PEGylated in a classical manner to enhance its biocompatibility and non-specific repellency, improving its stability and prolonging the half-life during the circulation 30. Without PEGylation, the precipitate component could be found in the final reaction product. Unlike the traditional chemical synthesis, Gd@Hb^Ce6-PEG^ nanoparticles were synthesized *via* a green and biomimetic approach based on hemoglobin's multiple roles. It functions as a biological template, stabilizer for the nanoparticle formation, and substrate for the loading of Ce6 *via* the hydrophobic domains. The synthetic principle of Gd@Hb^PEG^ nanoparticles is mainly attributed to the high affinity of Hb's amino acid residues with Gd^3+^ metal ions and the constrained effect of Hb, providing reaction sites for Gd-containing nanoparticle formation and also inhibits their overgrowth into large particles 22.

**Figure 1:**
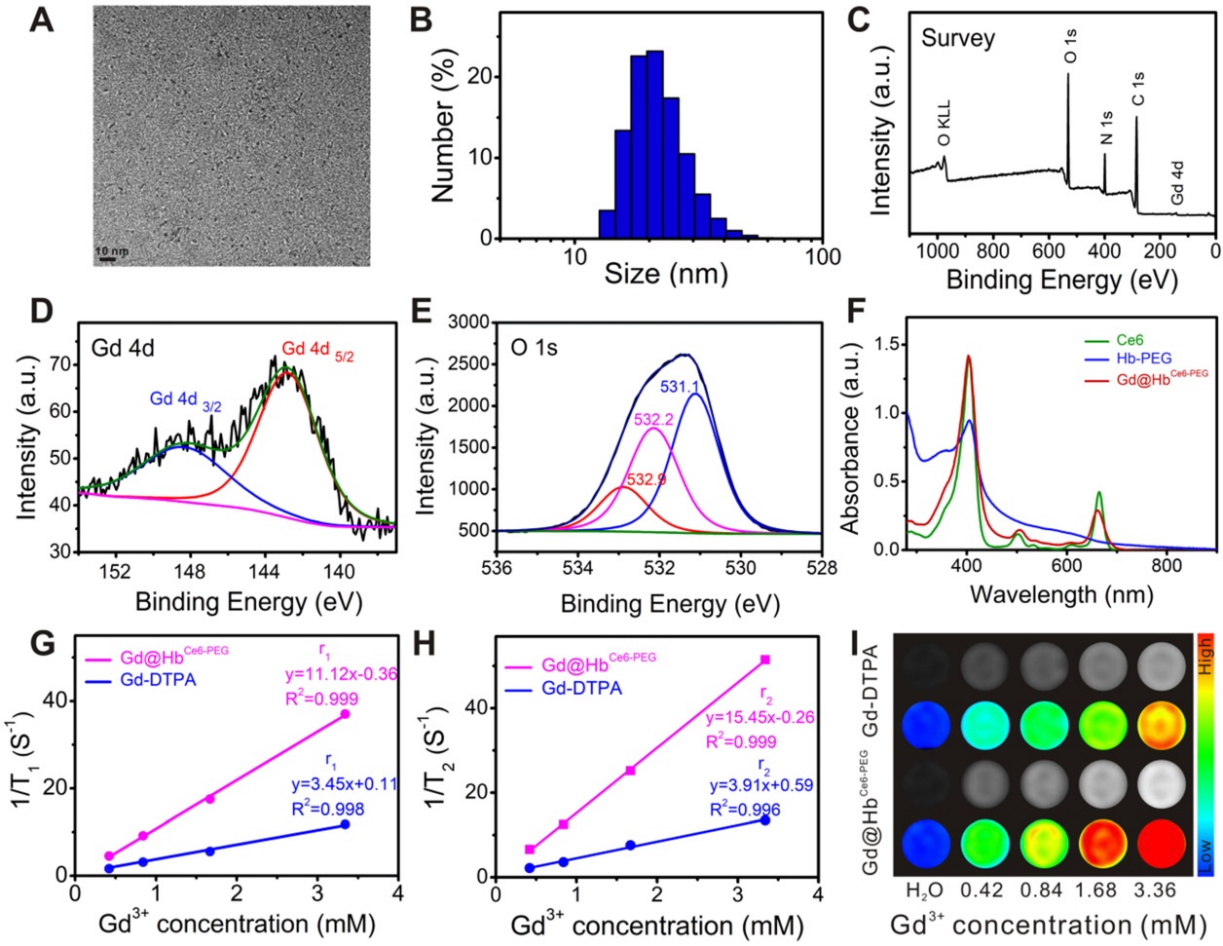
** Characterizations of Gd@Hb^Ce6-PEG^.** (**A**) TEM image of Gd@Hb^Ce6-PEG^. (**B**) Size distribution of Gd@Hb^Ce6-PEG^ determined by dynamic light scattering (DLS). (**C**) XPS survey spectrum of Gd@Hb^Ce6-PEG^. (**D, E**) XPS spectra of Gd 4d (D) and O 1s (E) in Gd@Hb^Ce6-PEG^ structure with the corresponding fitting curves analyzed by XPS Peak 4.1 software. (**F**) UV-vis absorbance spectra of Ce6, Hb-PEG, and Gd@Hb^Ce6-PEG^. (**G, H**) Longitudinal (*r_1_*) (G) and transverse (*r_2_*) (H) relaxation rates obtained from solutions of Gd@Hb^Ce6-PEG^ nanoparticles and the clinical contrast agent Magnevist (Gd-DTPA). (**I**) *In vitro* T_1_-weighted images of Gd@Hb^Ce6-PEG^ and Gd-DTPA.

The main physicochemical properties of Gd@Hb^Ce6-PEG^ were investigated by transmission electron microscopy (TEM), showing a uniform spherical structure with an ultrasmall size of about 4.0 nm (Figure 1A). The ultrasmall size mainly benefited from the protein-mediated constrained synthesis process 31, 32. The small-sized probes favor tumor theranostics since they can be cleared from the body *via* kidneys after imaging and therapy, as demonstrated below. The hydrodynamic size (HD) of the Gd@Hb^Ce6-PEG^ was *ca.* 21.0 nm with a good size distribution (PDI: 0.126) (Figure 1B), which was larger than the TEM result due to Hb-PEG encapsulation but difficult to be observed in Figure 1A. The size of Gd nanoparticles could be tuned by adjusting the ratio of raw materials (Figure S1, Supporting Information). The Gd@Hb^Ce6-PEG^ nanoparticles showed favorable stability in deionized water, PBS, fatal bovine serum (FBS) (Figure S2), sodium chloride with various ionic strengths, and various pH buffer solutions with slight fluctuations in their HDs (Figure S3). These results indicated good colloidal stability of Gd@Hb^Ce6-PEG^ nanoparticles due to the strong of hemoglobin encapsulation.

X-ray photoelectron spectroscopy (XPS) analysis was conducted to determine the elemental composition and chemical states of the synthesized material. Figure 1C demonstrates the co-existence of Gd and O elements. Their specific chemical states in the Gd@Hb^Ce6-PEG^ nanoparticles were confirmed by analyzing the Gd (4d) and O (1s) peaks. As shown in Figure 1D, the peaks of binding energies located at 142.9 eV and 148.2 eV in the Gd 4d spectrum represented Gd (OH)_3_ and Gd_2_O_3_, respectively. The peaks of binding energies located at 531.1 eV and 532.2 eV corresponded to the oxygen in Gd_2_O_3_ and Gd(OH)_3_, respectively. The 532.9 eV peak was attributed to the oxygen in -OH and -COOH of Hb molecule (Figure 1E) 22, 33-35. As shown in the UV-vis absorption spectrum in Figure 1F, the absorption peaks centered at 504 nm and 656 nm in Gd@Hb^Ce6-PEG^ were the characteristic Ce6 peaks, indicating the successful loading of Ce6. The release of Ce6 from Gd@Hb^Ce6-PEG^ nanoparticles was determined *in vitro* by investigating the UV-vis spectra of the filtrate obtained at different time points before and after ultrafiltration. The results (Figure S4) showed that the cumulative Ce6 release rate increased within 1 h and then saturated, indicating that Ce6 bound to proteins was relatively stable. These results collectively showed a successful synthesis of Gd@Hb^Ce6-PEG^ by using hemoglobin.

To determine the T_1_-weighted imaging performance of Gd@Hb^Ce6-PEG^, longitudinal and transverse relaxation rates (*r_1_* and *r_2_*) were evaluated by measuring 1/T_1_ and 1/T_2_ (s^-1^) slopes of the corresponding Gd^3+^ concentrations. According to previous studies 36, 37, the proton retention lifetime (τ_m_), the number of coordinated water molecules (q) bound to the paramagnetic metal ions, and the rotation correlation time (τ_R_) are the three crucial factors that determine the T_1_ relaxation rate. Thus, acquiring a longer rotation correlation time or a larger number of bound water molecules effectively improves the longitudinal relaxation rate. For Gd-DTPA used in clinical practice, only one coordination site is utilized to form a coordination bond with a water molecule, and the molecular rotational correlation time(τ_R_) of Gd^3+^ small molecule chelate compounds is much shorter compared to that of the Gd-based macromolecules 38.

In this study, the proper size of 4.0 nm brought about a larger number of bound water molecules (q) and the encapsulation of biomacromolecule offered a longer rotation correlation time (τ_R_), resulting in increased relaxivity. As displayed in Figure 1G and 1H, the longitudinal and transverse proton relaxation rates (*r_1_* and *r_2_*) were 11.12 mM^-1^•s^-1^ and 15.45 mM^-1^•s^-1^, respectively, and the value of *r_2_/r_1_* ratio was calculated to be 1.39, indicating the suitability of Gd@Hb^Ce6-PEG^ as a T_1_-weighted imaging contrast agent 38. The MRI stability Gd@Hb^Ce6-PEG^ was good in deionized water, PBS, and FBS, without obvious fluctuations in T_1_ relaxation time (Figure S5). To further evaluate the Gd ions dissociation quantitatively, we measured the Gd concentration by ICP-AES before and after ultrafiltration. The results are summarized in the supporting information as Table S1, showing the leakage percentage of Gd ions to be less than 0.1% after 1 day and 7 days. These results indicated that the synthesized Gd@Hb^Ce6-PEG^ nanoprobes were stable without leakage of Gd ions and were suitable for *in vivo* applications. The *r_1_* of Gd@Hb^Ce6-PEG^ was almost three times higher than the clinically-used Gd-DTPA (*r_1_* = 3.45 mM^-1^•s^-1^). Furthermore, the *in vitro* T_1_-weighted images of Gd@Hb^Ce6-PEG^ and Gd-DTPA (Figure 1I) and their corresponding quantified MR signal intensities (Figure S6) confirmed that Gd@Hb^Ce6-PEG^ nanoparticles could produce higher T_1_-weighted signal intensity than Gd-DTPA.

### Oxygen-carrying ability/release and singlet oxygen generation capability of Gd@Hb^Ce6-PEG^

**Figure 2:**
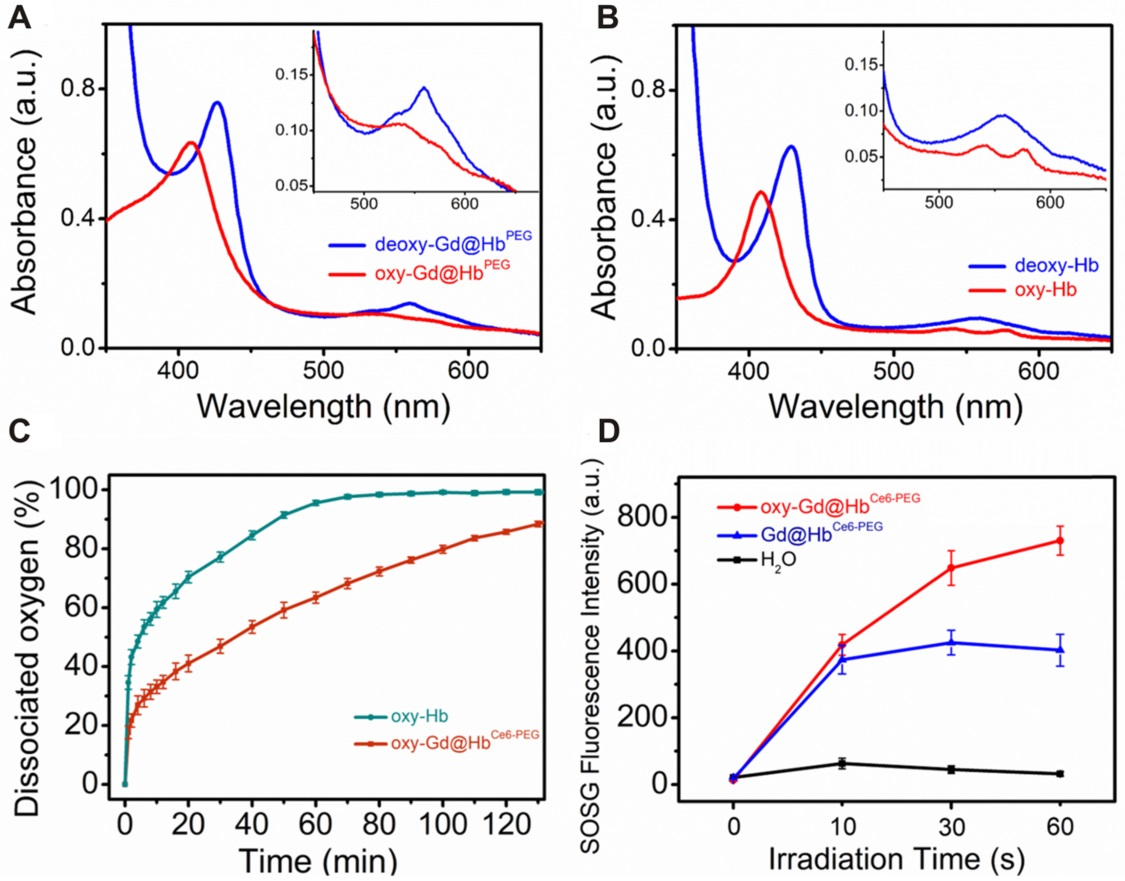
** Oxygen-carrying ability/release and ^1^O_2_ generation capability study of Gd@Hb^Ce6-PEG^.** (**A, B**) UV-vis absorbance spectra of (A) Gd@Hb^PEG^ and (B) pure Hb in the oxygen-free and oxygen saturation conditions. The inset image shows details of the absorption between 450 nm and 660 nm. (**C**) Oxygen-dissociation curves of oxy-Hb and oxy-Gd@Hb^Ce6-PEG^. (**D**) ^1^O_2_ generation of oxy-Gd@Hb^Ce6-PEG^ and Gd@Hb^Ce6-PEG^ measured by the SOSG fluorescence intensity under 660 nm laser illumination (0.6 mW/cm^2^).

Using hemoglobin, a compact structure of Gd@Hb^Ce6-PEG^ with integrated functional moieties was biomimetically synthesized. As mentioned earlier, besides the biomimetic chemistry, another important advantage of using hemoglobin in this study was to deliver oxygen into tumor tissues. To eliminate the interference of the absorption spectrum of Ce6, Gd@Hb^PEG^ nanoparticles were prepared and the UV-vis spectra were applied to examine the ability of de-oxygenation and oxygenation of the Gd@Hb^PEG^ (Figure 2A) and pure Hb (Figure 2B). After the addition of sodium dithionite, Gd@Hb^PEG^ exhibited two maximum absorption peaks located at 428 nm and 565 nm, denoting 100% de-oxygenation (deoxy-Gd@Hb^PEG^) 39. When pure oxygen gas was allowed to draw into the sample chamber, three maximum peaks located at 412 nm, 540 nm, and 575 nm were observed, of which bimodal peaks (540 nm and 575 nm) were the characteristic absorption peaks of oxygenated Gd@Hb^PEG^. For comparison, the oxygenation process of pure Hb was conducted and its spectral changes were found to be similar to that of Gd@Hb^PEG^, demonstrating the oxygen-carrying ability of Hb after biomimetic synthesis. Also, the UV-vis spectra and T_1_ relaxation times displayed no significant fluctuations after oxygenation (Figure S7), indicating that the oxygenation of Hb exerted no obvious influence on Ce6 and Gd.

Furthermore, the oxygen-dissociation curve was determined to monitor the oxygen release behavior of oxy-Gd@Hb^Ce6-PEG^ and oxy-Hb. As shown in Figure 2C, the half-dissociation time value measured for oxy-Hb was about 6.2 min, consistent with a previous study 40. In contrast, oxy-Gd@Hb^Ce6-PEG^ showed slower oxygen release behavior, with 34 min of the half-dissociation time of oxy-Gd@Hb^Ce6-PEG^. This difference in release kinetics could be mainly attributed to the structure change of Hb with improved stability.

To observe the photodynamic effect of oxy-Gd@Hb^Ce6-PEG^ and Gd@Hb^Ce6-PEG^, the amount of singlet oxygen (^1^O_2_) generation was evaluated by using the singlet oxygen sensor green (SOSG) probe. As Figure 2D and Figure S8 display, after laser irradiation for 1 min, the SOSG fluorescence intensity of oxy-Gd@Hb^Ce6-PEG^ group was two times higher than Gd@Hb^Ce6-PEG^ under the same condition, indicating enhanced ^1^O_2_ generation due to more oxygen involvement.

### Cellular uptake, cytotoxicity, and photodynamic effects of Gd@Hb^Ce6-PEG^ nanoparticles

Cellular uptake of Gd@Hb^Ce6-PEG^ was observed by using a confocal laser scanning microscope (CLSM). Figure S9 shows the confocal images of 4T1 breast cancer cells treated with 30 μg/mL Gd@Hb^Ce6-PEG^ nanoparticles for 1 h, 2 h, and 4 h. The fluorescence signals were observed in 4T1 breast cancer cells, indicating efficient internalization of Gd@Hb^Ce6-PEG^ nanoparticles.

**Figure 3:**
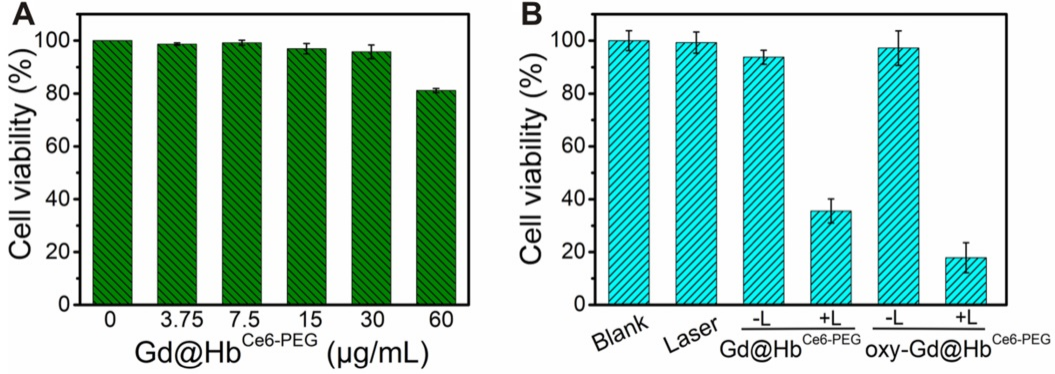
** Cytotoxicity and photodynamic effects of Gd@Hb^Ce6-PEG^** (**A**) 4T1 cell viability after treatment with different concentrations of Gd@Hb^Ce6-PEG^ nanoparticles (0-60 µg/mL). (**B**) 4T1 cell viability with Gd@Hb^Ce6-PEG^ or oxy-Gd@Hb^Ce6-PEG^ (30 µg/mL each) nanoparticles and with or without 660 nm laser irradiation. L, Laser.

Subsequently, CCK-8 assay was performed to measure the cytotoxicity of Gd@Hb^Ce6-PEG^ on 4T1 cells. As displayed in Figure 3A, negligible effect was noticed on viability of 4T1 cells incubated with Gd@Hb^Ce6-PEG^ in the absence of light at tested concentrations (0, 3.75, 7.5, 15, 30, and 60 μg/mL) for 24 h at 37 °C, 5% CO_2_. The cell viability was about 80% at 60 μg/mL, indicating low toxicity of Gd@Hb^Ce6-PEG^ nanoparticles. The 4T1 cell viability following treatment with 660 nm laser irradiation at different concentrations of Gd@Hb^Ce6-PEG^ was also analyzed (Figure S10). Results showed more efficient PDT efficacy on 4T1 cells with increasing concentrations of Gd@Hb^Ce6-PEG^.

Next, the *in vitro* photodynamic therapy of oxy-Gd@Hb^Ce6-PEG^ was examined in 4T1 cells. The viability of 4T1 cells incubated with 30 μg/mL Gd@Hb^Ce6-PEG^ and oxy-Gd@Hb^Ce6-PEG^ and 660 nm laser irradiation decreased to 34% and 18%, respectively, indicating efficient killing of 4T1 cells (Figure 3B). Compared with Gd@Hb^Ce6-PEG^, oxy-Gd@Hb^Ce6-PEG^ showed an enhanced photodynamic effect with the same laser irradiation condition, mainly attributed to the oxygen-carrying capability of oxy-Gd@Hb^Ce6-PEG^. These results indicated the low dark toxicity but good photodynamic effects of oxy-Gd@Hb^Ce6-PEG^ on 4T1 breast cancer cells.

### *In vivo* MR imaging guidance

**Figure 4:**
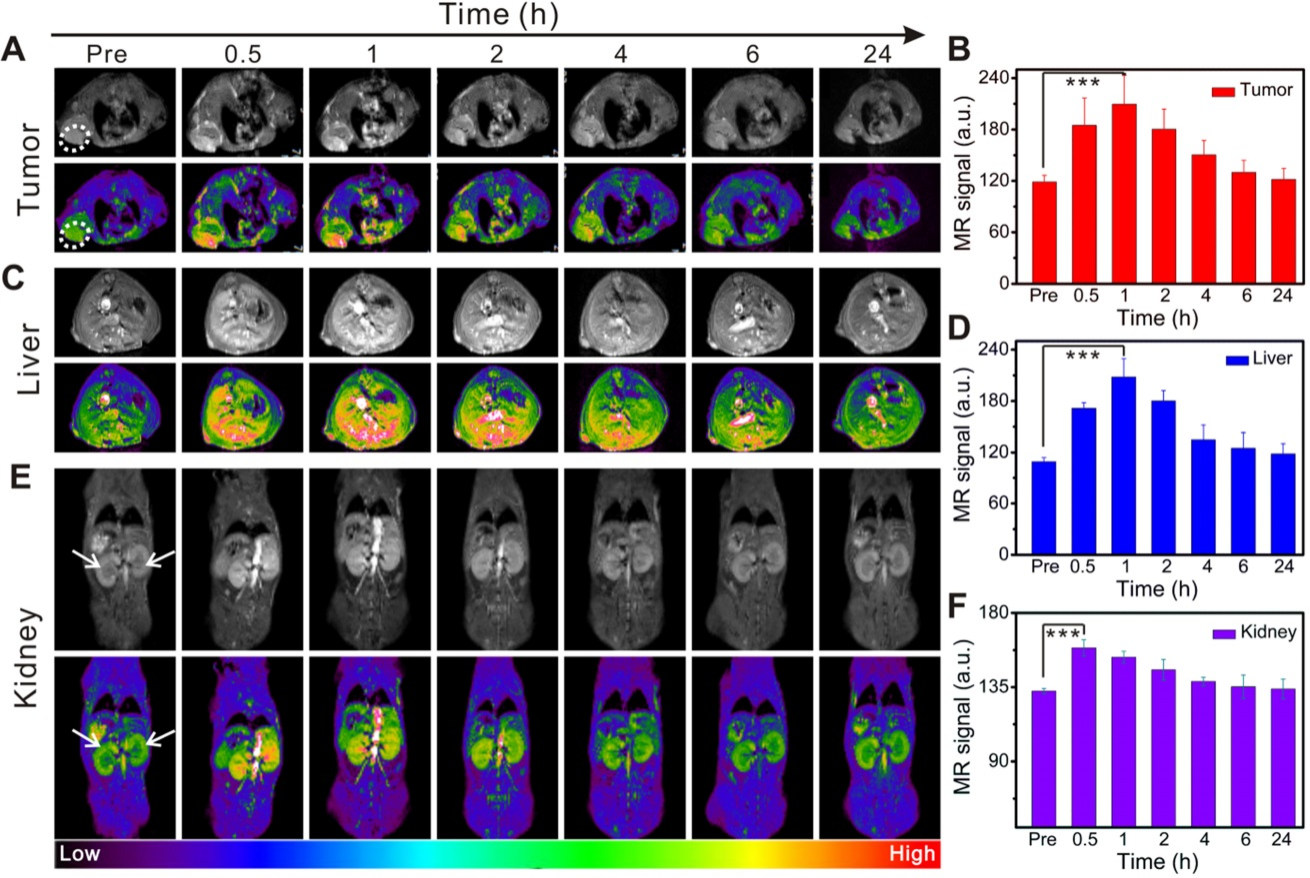
***In vivo* time-dependent MR imaging of the tumor, liver, and kidneys in 4T1 tumor-bearing mice before and after intravenous injection of Gd@Hb^Ce6-PEG^.** (**A, C, E**) *In vivo* T_1_-weighted MR images of the tumor (A, white circle), liver (C), and kidneys (E, white arrow). Quantified MR signal intensity of 4T1 tumor (**B**), liver (**D**), and kidneys (**F**).

As described above, we verified the oxygen-carrying/release capability of Gd@Hb^Ce6-PEG^ and its PDT effect on tumor cells. PDT is a promising treatment strategy for superficial and lumen tumors but it is difficult to find a therapeutic window due to the lack of clear imaging guidance. In this study, the paramagnetic Gd element was selectively incorporated into the nanoparticles to realize MR imaging-guided PDT. MR images (Figure 4A) and the corresponding quantified intensities (Figure 4B) showed a gradual increase in tumor signal intensity after intravenous injection and a decrease beginning at 2 h post-injection. The maximal tumor uptake occurred at 1 h post-injection. The tumor uptake of Gd@Hb^Ce6-PEG^ was demonstrated in MR imaging, displaying a remarkably higher signal intensity than in the Gd-DTPA group (Figure S11). The dynamic process of MR signal intensity changes was further revealed by MIP (maximum intensity projection) images, reflecting the presence of blood vessels in the tumor (Figure S12).

Besides imaging the tumor sites, MR imaging of the liver and kidneys was acquired to dynamically assess Gd@Hb^Ce6-PEG^ nanoparticles' biodistribution *in vivo*. Figure 4C shows a gradual increase in the MR signal intensity of liver, reaching the maximum at 1 h after injection, followed by a gradual decrease. At 24 h post-injection, the signal decreased, almost to pre-injection levels, as quantified in Figure 4D. The signals in kidneys were very similar to the liver (Figure 4E and 4F). These *in vivo* imaging data showed hepatic and renal clearance pathways of Gd@Hb^Ce6-PEG^ nanoparticles from the body within 24 h. The renal clearance was attributed to the ultrasmall size of the synthesized Gd@Hb^Ce6-PEG^, conducive to reducing exposure time/dosage of nanoparticles in the liver. Nanoparticles synthesized by traditional chemical methods usually accumulate in the liver for more than 24 h, resulting in long-term accumulation and presenting potential hepatotoxicity 41, 42. Distinctly different from most previously reported nanoparticles, this biomimetic synthesis of Gd@Hb^Ce6-PEG^ nanoparticles was beneficial for reducing liver toxicity.

**Figure 5:**
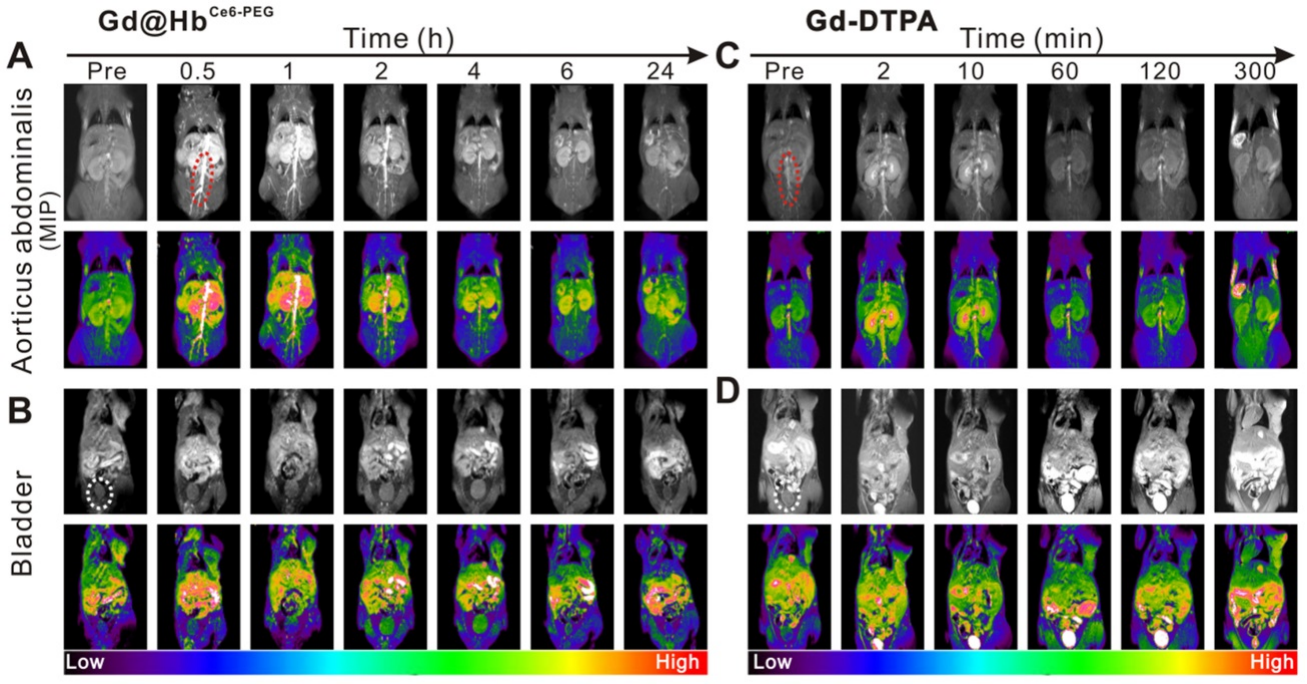
***In vivo* MR angiography and MRI of the bladder in mice.** (**A, C**) MIP images of aorticus abdominalis (red ellipse) with intravenous injection of Gd@Hb^Ce6-PEG^ nanoparticles (A) and Gd-DTPA (C). (**B, D**) T_1_-weighted MR imaging of the bladder (white ellipse) of mice with intravenous injection of Gd@Hb^Ce6-PEG^ nanoparticles (B) and Gd-DTPA (D). MIP, maximum intensity projection.

The circulation half-life of Gd@Hb^Ce6-PEG^ was determined to be ~1 h (Figure S13) by measuring Gd content with ICP-AES. Furthermore, angiography of Gd@Hb^Ce6-PEG^ was performed in mice. As is evident from Figure 5A and 5C, the MIP images of aortic abdominalis (red ellipse) reflected the imaging time window difference with Gd@Hb^Ce6-PEG^ showing much longer imaging time than Gd-DTPA in blood vessels. This difference could mainly be attributed to the particle size of the injected agent. Gd-DTPA is a small molecule that can be rapidly cleared from the body *via* the renal pathway 43, while Gd@Hb^Ce6-PEG^ is sub-10 nm in size that favors its circulation in blood.

Besides the size, hemoglobin, an endogenous protein functions like an invisible cloak on nanoparticles, and escapes capturing by the liver. Figure 5B and Figure S14A show the process by which the contrast agent excretes into the bladder *via* the renal pelvis route during the circulation. The imaging time window of the abdominal aorta in mice receiving intravenous injection of Gd@Hb^Ce6-PEG^ nanoparticles was wider and less contrast agent was excreted in the renal pelvis at 1 h after the injection. For mice injected with Gd-DTPA, the imaging time of the aorticus abdominalis was much shorter with a weaker signal intensity, and most of the nanoparticles were excreted into the renal pelvis and bladder at 2 min after the injection (Figure 5D and Figure S14B). Overall, compared with the clinical contrast agent (Gd-DTPA), the signal intensity in the tumor increased significantly after intravenous injection of Gd@Hb^Ce6-PEG^ particles, possibly benefiting from the enhanced permeability and retention effect (EPR) of nanomaterials and its improved relaxivity. More importantly, the imaging time window was extended from a few min to 1 h-2 h, providing more time for guiding treatment and ultimately improving the therapeutic effect.

### *In vivo* measurement of tumor oxygenation

**Figure 6:**
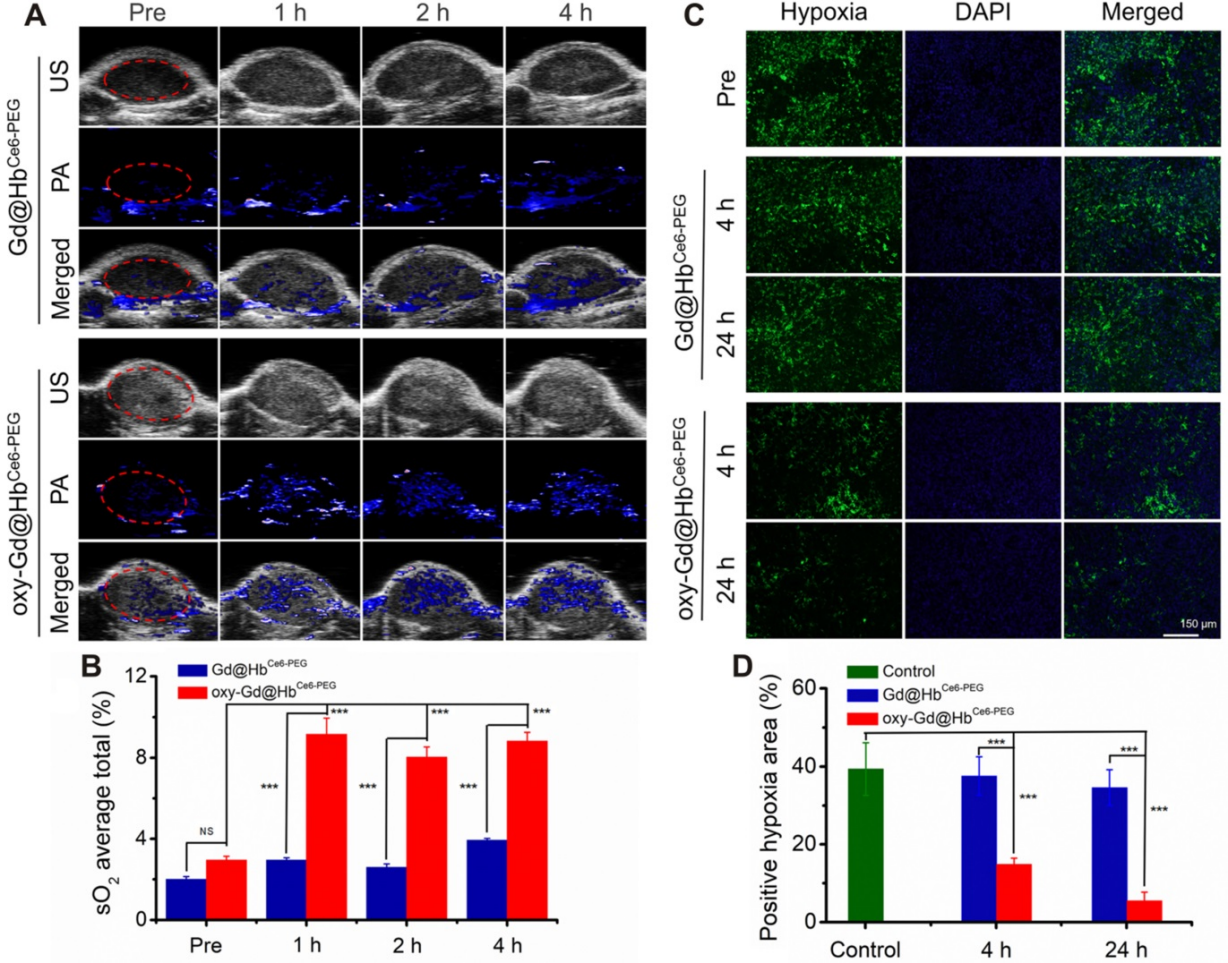
***In vivo* assessment of tumor oxygenation in mice with 4T1 tumor.** (**A**) PA images of the tumor sites recorded in the oxy-hemo mode before and at 1 h, 2 h, and 4 h post intravenous injection of Gd@Hb^Ce6-PEG^ or oxy-Gd@Hb^Ce6-PEG^ nanoparticles. (**B**) Corresponding semiquantitative analysis of overall tumor oxygenation saturation (sO_2_ average total) at different time points. (**C**) Images of immunofluorescence staining collected from mice tumor tissues with intravenous injection of Gd@Hb^Ce6-PEG^ or oxy-Gd@Hb^Ce6-PEG^ nanoparticles. The hypoxic areas and cell nuclei were stained with anti-HIF-1α antibody (green) and DAPI (blue). (**D**) Corresponding semiquantitative analysis of the percentages of positive hypoxic areas by calculating HIF-α protein expression density in the image. PA, photoacoustic imaging; US, ultrasound imaging.

After demonstrating the oxygen-carrying and release capacity and tumor targeting of Gd@Hb^Ce6-PEG^, we evaluated the oxygen delivering capability of oxy-Gd@Hb^Ce6-PEG^ for improving the overall oxygen saturation in the tumor area by using photoacoustic (PA) imaging *in vivo*. As shown in the PA images taken under the oxy-hemo mode, the PA signal intensity in the tumor area increased after intravenous injection of oxy-Gd@Hb^Ce6-PEG^ nanoparticles (Figure 6A). However, only a slight effect on the tumor oxygenation level was observed in the Gd@Hb^Ce6-PEG^ nanoparticle-treated mice. The semiquantitative analysis was performed for the region of interest (ROI) in tumor sites by PA imaging. The results showed that the overall tumor oxygenation saturation (sO_2_ average total) level increased from *ca.* 2.9% before the injection of oxy-Gd@Hb^Ce6-PEG^ nanoparticles to about 8.7% at 2-4 h post-injection (Figure 6B).

Hypoxia is one of the important features of the tumor microenvironment and is directly related to the expression of HIF-1α protein 44. To determine the ability of oxy-Gd@Hb^Ce6-PEG^ to alleviate tumor hypoxia, immunofluorescence staining was performed for evaluating the change of hypoxic state (expression of HIF-1α protein) in the tumor following intravenous injection of oxy-Gd@Hb^Ce6-PEG^ nanoparticles. HIF-1α immunofluorescence staining displayed decreased tumor hypoxia in the tumor slices collected at 4 h post-injection of oxy-Gd@Hb^Ce6-PEG^ compared with the images obtained from the control group mice (Figure 6C). In contrast, mice injected with Gd@Hb^Ce6-PEG^ showed minimal hypoxic state change in the tumor. Compared with the control group, the percentage of hypoxic tumor area decreased from *ca*. 39% to *ca.*14% and *ca.* 6% at 4 h and 24 h post-injection of oxy-Gd@Hb^Ce6-PEG^ (Figure 6D). However, no obvious change was observed in the tumor slices of mice injected with Gd@Hb^Ce6-PEG^. Collectively, these data confirmed that oxy-Gd@Hb^Ce6-PEG^ could deliver oxygen for tumor oxygenation and alleviate tumor hypoxia *in vivo*.

### *In vivo* PDT and morphological MR imaging evaluation of therapeutic efficacy

**Figure 7:**
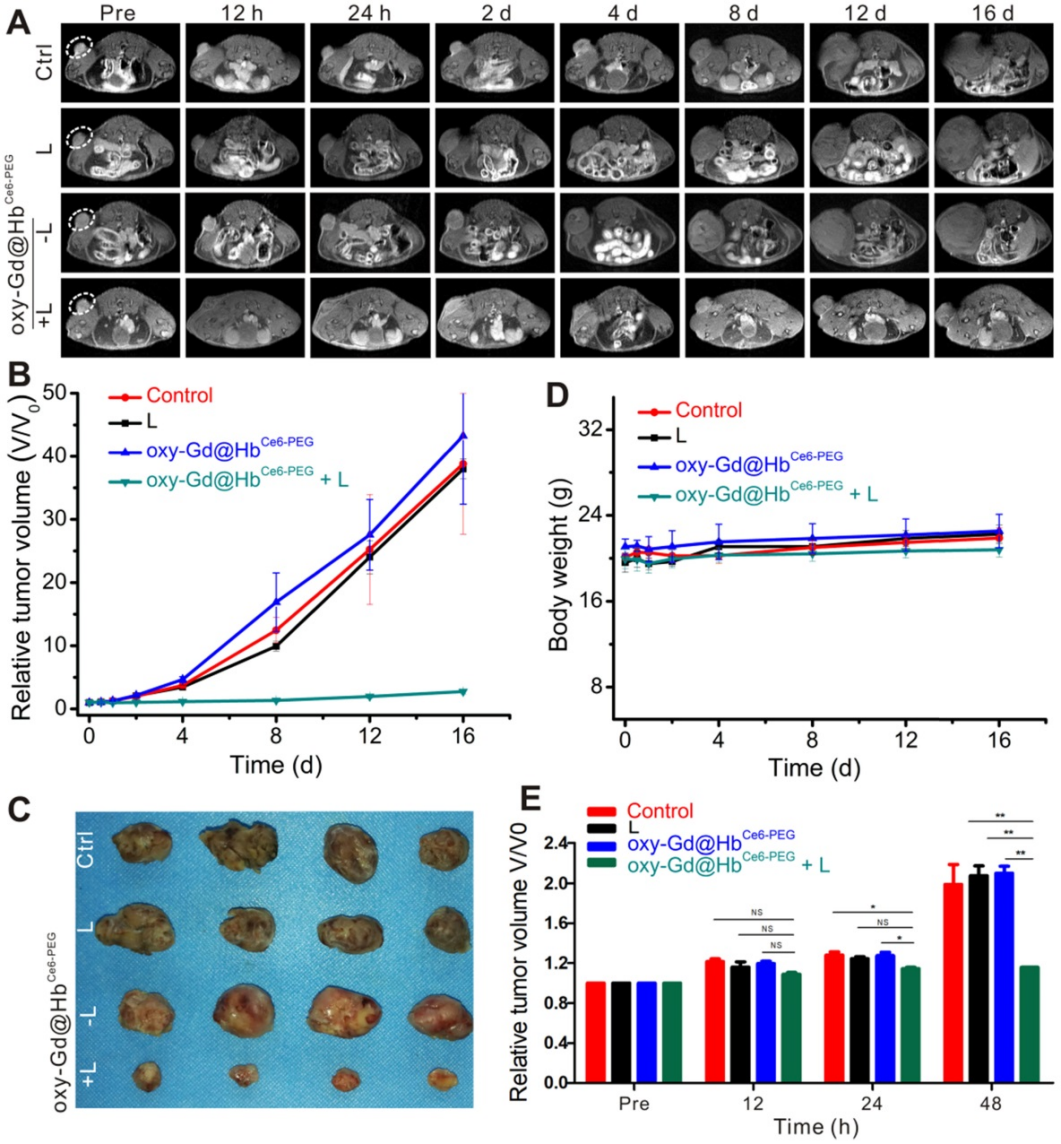
***In vivo* PDT in tumor-bearing mice and MR imaging-based morphology evaluation of therapeutic efficacy.** (**A**) T_1_-weighted MR imaging of mice in four groups before and after respective treatments. (**B**) Relative tumor volume changes of mice in four groups during the experiment. (**C**) Digital pictures of tumors obtained from the four groups after respective treatments. (**D**) Body weight changes of mice in four groups during the experiment. (**E**) MR imaging evaluation of therapeutic efficacy based on morphological changes. The therapeutic effect could be determined on day 2 after treatment. Data are presented as the mean ± standard deviation. (*P< 0.05; **P< 0.01; ***P< 0.001; n = 4). L, Laser.

We exposed 4T1 tumor-bearing mice to PDT with 660 nm laser irradiation for 10 min after 2 h of intravenous administration of oxy-Gd@Hb^Ce6-PEG^ nanoparticles. The tumor size was accurately measured during the treatment by MR imaging to assess therapeutic efficacy. As displayed in Figures 7A, 7B, and 7C, the tumor volumes of mice in the control, laser irradiation only, and oxy-Gd@Hb^Ce6-PEG^ nanoparticle groups were significantly increased during treatment, while tumor growth was inhibited in the oxy-Gd@Hb^Ce6-PEG^+laser irradiation group. Also, there were no changes in the body weights of mice in the experimental groups during the treatment (Figure 7D), indicating no obvious adverse systemic effects after PDT. Statistical analysis of the data using MR imaging of tumor morphology indicated therapeutic efficacy at day 2 after treatment (Figure 7E).

### Functional MRI-based early evaluation of therapeutic efficacy

To evaluate the efficacy of PDT, functional MRI was carried out. Estimating the gross tumor volume by measuring the physical diameters of the tumor with calipers is a common method for evaluating PDT's efficacy 45, 46. However, this method cannot determine morphological changes of tumors. In clinical studies, MR imaging-based evaluation is usually utilized to assess the curative effect of anti-tumor treatment. Monitoring the morphological changes of the tumor provides clinicians with more precise feedback for tumor treatment 47, 48. However, the morphological changes of tumors are not always sensitive to the treatment in the early term.

Diffusion-weighted imaging (DWI) is a functional MR imaging that reflects changes in tissue microstructure by detecting the extent and direction of localized diffusion of water molecules in living tissues. This method can accurately reflect the changes in the body tissues' microscopic spatial composition and the functional status of water exchange between various tissue components under pathophysiological conditions 49. The pathophysiological intracellular changes occurring within tumor tissue after treatment reflected by DWI can indicate the early changes in the tumor's interior prior to visible changes in tumor morphology or size 50. The diffusion coefficient measured by DWI can be quantified by the apparent diffusion coefficient (ADC) parameter 49. ADC value ​​can reflect the tissue-specific diffusion capacity and detect early changes in tumor cell structure after treatment 50. An increase in the ADC value indicates that the treatment of malignant tumors is effective and has been confirmed in a variety of malignant tumors, including breast cancer 51. PDT induces tumor cell necrosis or apoptosis within the first 24 h of treatment 52. These microscopic structural changes cause a limited decrease in the diffusion motion of water molecules, resulting in an increased ADC value. On the contrary, when the tumor is in the progressive stage without the drug intervention, the ADC value gradually decreases with time as the tumor cells proliferate 50.

**Figure 8:**
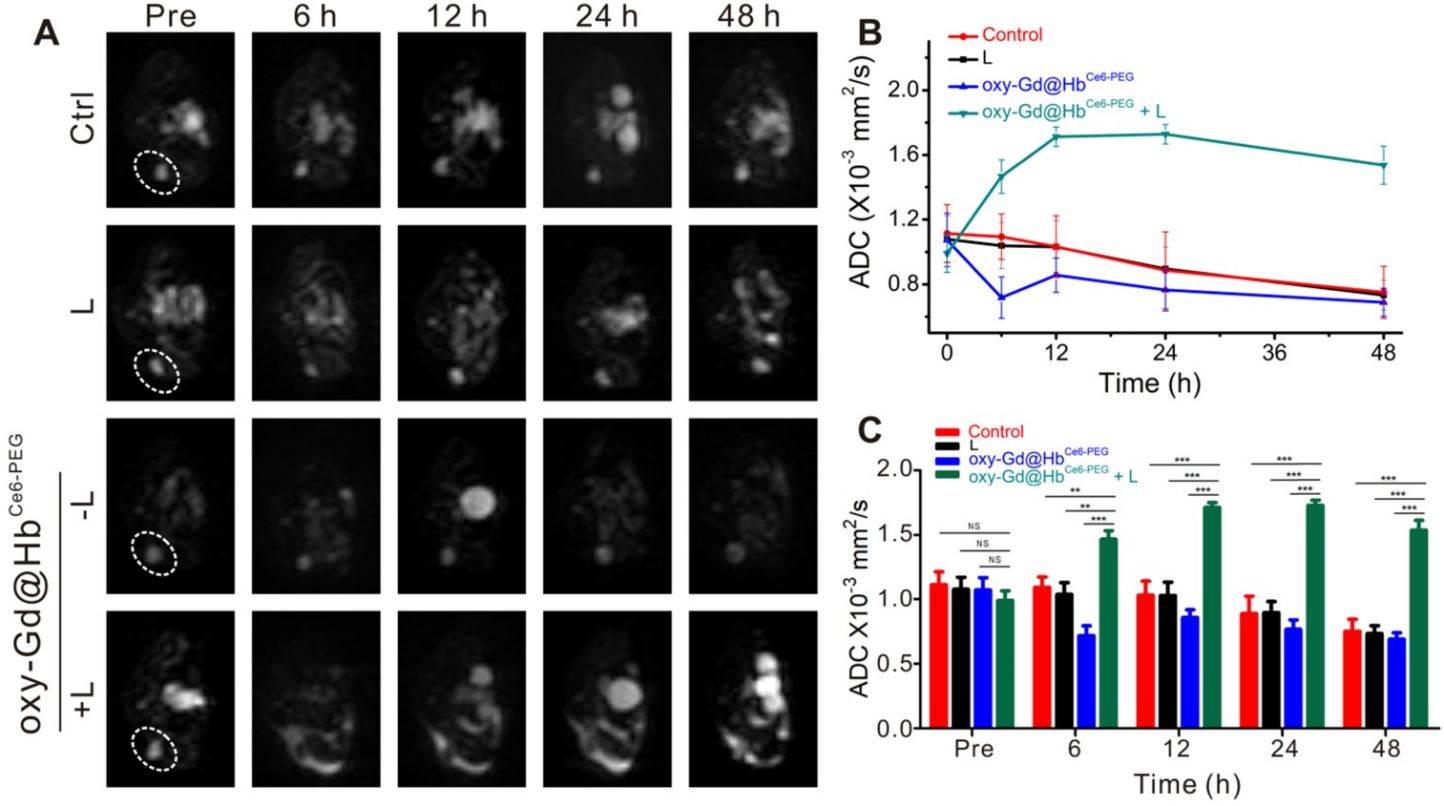
** DW imaging for early evaluation of therapeutic efficacy of PDT.** (**A, B**) DW imaging with b value for 1000 s/mm^2^ (A) and the corresponding changes of ADC values of lesions (B) in four groups during the first 48 h of treatment. (**C**) Quantification analysis of ADC values for early evaluation. Data are presented as the mean ± standard deviation. (*P< 0.05; **P< 0.01; ***P< 0.001; n = 4). L, Laser.

As shown in Figure 8A, the lesion areas of four groups present high signal intensity on the DWI map before treatment. During the treatment observation time, the lesion areas of mice from the groups treated with saline, laser irradiation, or oxy-Gd@Hb^Ce6-PEG^ nanoparticles showed high signal intensity on the DWI map and a gradual decrease of ADC values (Figure 8A and 8B). In the experimental group, however, the lesion areas consistently showed low intensity on DWI map after treatment and the ADC values were higher than pretreatment. Statistical analysis showed that, compared with the control group, the ADC values of the experimental group at different time points were significantly higher after treatment (Figure 8C). Using ADC values, the noticeable therapeutic efficacy of oxy-Gd@Hb^Ce6-PEG^ nanoparticles-based PDT was quantitatively confirmed at 6 h post-PDT, much earlier than that (day 2 post-PDT) evaluated by structural MR imaging (Figure 7E).

### *In vivo* toxicology analysis

Routine blood tests, biochemistry parameters, and H&E staining were performed at day 1 and day 14 post injection of Gd@Hb^Ce6-PEG^ nanoparticles (Gd dose: 0.11 mmol/kg) to examine the safety. As shown in Figure S15, no or slight changes were observed in the main parameters of the routine blood test, including red blood cells (RBC), mean corpuscular volume (MCV), red blood cell volume distribution width (RDW), hematocrit (HCT), reticulocyte (RET), hemoglobin (HGB), mean corpuscular hemoglobin (MCH), mean corpuscular hemoglobin concentration (MCHC), white blood cells (WBC), and platelets (PLT). After intravenous injection of Gd@Hb^Ce6-PEG^ nanoparticles, biochemistry blood tests, including alanine aminotransferase (ALT), aspartate aminotransferase (AST), alkaline phosphatase (ALP), total protein (TP), albumin (ALB), globulin (GLOB), A/G (ALB/GLOB), and blood urea nitrogen (BUN) indicated no or slight damage to the function of liver, kidneys and hematopoietic system.

H&E staining of major mouse organs was also conducted to determine the *in vivo* toxicity of Gd@Hb^Ce6-PEG^ nanoparticles. As displayed in Figure S16, compared with the control group (physiological saline injection), slightly reduced numbers of spleen lymphocytes were observed at day 1 and day 14 after intravenous injection of Gd@Hb^Ce6-PEG^ and no obvious inflammatory lesions or tissue damage could be detected in other organ tissues.

## Conclusion

We synthesized a hemoglobin-mediated biomimetic of paramagnetic nanostructure for tumor oxygenation and improved therapeutic outcomes of PDT. We demonstrated that hemoglobin could maintain tumor oxygenation activity in the Gd nanoparticles after loading the Ce6 photosensitizer. Thus, Gd@Hb^Ce6-PEG^ nanoparticles could simultaneously deliver oxygen and a photodynamic agent to the tumor tissues. The MR imaging moiety of Gd@Hb^Ce6-PEG^ showed good tumor-targeted accumulation and provided precise guidance for PDT. The hemoglobin moiety of Gd@Hb^Ce6-PEG^ delivered oxygen and overcame tumor hypoxia in tumor-bearing mice, as demonstrated by PA imaging and HIF-1α protein expression analysis. As expected, Gd@Hb^Ce6-PEG^ enabled good tumor-specific PDT by ameliorating tumor hypoxia together with MRI guidance. Furthermore, the therapeutic efficacy could be quantified with DWI at 6 h post-PDT.

To the best of our knowledge, our study is the first demonstration of biomimetic synthesis of ultrasmall paramagnetic Gd nanostructures on endogenous human Hb. Also, integration of Ce6 photosensitizers could be realized for MR imaging-guided oxygen-supplying PDT of tumors. Significantly, the design of the nanostructures did not require complicated chemical synthesis and the raw materials, including a human endogenous protein, were biocompatible and non-toxic. We believe that the method used here would find broad applications for fabricating advanced probes for biomedical uses.

## Experimental

### Materials

Human hemoglobin (Hb), gadolinium chloride hexahydrate (GdCl_3_·6H_2_O), and sodium dithionite (Na_2_S_2_O_4_) were obtained from Sigma Aldrich (USA). Chlorin e6 (Ce6) was acquired from J&K Scientific Ltd. (China). N-Hydroxylsuccinimide-derivatized methoxy-poly (ethylene glycol) (mPEG-NHS, M_W_ = 2000 Da) was purchased from Thermo Fisher Scientific (USA). Sodium hydroxide (NaOH), borate saline buffer (BBS, pH 8.2) and phosphate-buffered saline (PBS, pH 7.4) were obtained from Sinopharm. Cell Counting Kit-8 (CCK-8) was purchased from KeyGEN bioTECH (Nanjing, China).

### Biomimetic synthesis of Gd@Hb^Ce6-PEG^ nanoparticles

Synthesis of Hb-PEG bioconjugates: First, 62.5 mg of Hb powder and 20 mg of mPEG-NHS were dissolved in 8 mL and 1 mL of BBS. Next, the two solutions were mixed and magnetically stirred at 25 °C for 4 h. The resulting solution was transferred to a dialysis bag and dialyzed against PBS for 12 h to remove byproducts.

Synthesis of Gd@Hb^Ce6-PEG^: 150 μL of GdCl_3_·6H_2_O (100 mM) was added into the above Hb-PEG solution, followed by mixing with 12 mg of Ce6, which was dissolved in 1 mL of DMSO. Then, 150 μL of NaOH (1.0 M) was added to the above solution 3 min later. After the mixture was magnetically stirred at 37 °C for 5 h, the solution was collected into a dialysis bag and dialyzed against PBS for 6 h. Gd@Hb^PEG^ was prepared according to the same procedure but without the addition of Ce6.

The oxy-Gd@Hb^Ce6-PEG^ was prepared following a previously reported method 28, 29. Briefly, the sodium ascorbate (2-fold molar of Hb) was added to the Gd@Hb^Ce6-PEG^ solution and sealed under N_2_ atmosphere to perform de-oxygenation process. After de-oxygenation, pure oxygen gas was allowed to run through the Gd@Hb^Ce6-PEG^ (deoxy-Gd@Hb^Ce6-PEG^) solution under a low flow rate to perform the oxygenation process.

### Characterization of nanoparticles

Transmission electron microscopy (TEM) was used to characterize the morphology and size of nanoparticles with an accelerating voltage of 200 kV. The X-ray photoelectron spectroscopy (XPS) studies were conducted to determine the elemental composition and chemical states of nanoparticles by using a PHI-5000 CESCA system (PerkinElmer). The hydrodynamic size and size distribution were monitored by dynamic light scattering (DLS) on a Malvern Zetasizer Nano ZS instrument. The UV-vis absorption spectrum of Gd@Hb^Ce6-PEG^ was recorded by using a Cary 50 spectrophotometer (VARIAN). The Gd content was measured by inductively coupled plasma-atomic emission spectroscopy (ICP-AES, Thermal Scientific, iCAP 7400).

### Ce6 release behavior study

The release of Ce6 from Gd@Hb^Ce6-PEG^ nanoparticles was determined *in vitro* by investigating the UV-vis spectra of the filtrate obtained at 656 nm at different time points before and after ultrafiltration. Subsequently, the cumulative Ce6 release curve versus time was obtained, and the solution was slowly stirred during the process. To evaluate the Gd dissociation *in vitro* quantitatively, we measured the Gd concentration by ICP-AES before and after ultrafiltration.

### Measurement of relaxivity and *in vitro* MR imaging study

The longitudinal (T_1_) and transverse (T_2_) proton magnetic relaxation times of Gd@Hb^Ce6-PEG^ were analyzed with a 1.41 T minispec mq 60 NMR Analyzer (Bruker) at 37 °C. The relaxation rates (*r_1_* and *r_2_*) were determined by measuring 1/T_1_ and 1/T_2_ (s^-1^) slopes to the corresponding Gd^3+^concentrations. For comparison, the relaxation times of Magnevist (Gd-DTPA) were also measured.

The SE (spin echo) T_1_-weighted MR images of Gd@Hb^Ce6-PEG^ and Gd-DTPA were obtained with a 3.0 T MRI scanner (Ingenia; Philips, The Netherlands) with an animal coil. The used scan parameters were set as follows: TR = 595 ms; TE = 20 ms; flip angle=90°; FOV = 120×120 mm; voxel size = 0.42×0.51×2.5 mm; slice gap = 0.8 mm; slice thickness = 2.5 mm; Total Scan Duration = 1 min 50 s.

### Oxygen-carrying capacity study

The capability of de-oxygenation and oxygenation of Gd@Hb^PEG^ were investigated by using a previously reported method 39, 53. First, the Gd@Hb^PEG^ (7 mg/mL) solution was de-oxygenated by sodium dithionite. After removing oxygen, the absorption spectrum of the solution was measured in the wavelength range 350 nm-700 nm. Then, the de-oxygenated solution was allowed to run with pure oxygen gas, followed by the measurement of the absorption spectrum of oxygenated Gd@Hb^PEG^ (oxy-Gd@Hb^PEG^) solution. Also, the oxygenation effect of Hb on Ce6 and Gd was evaluated by investigating UV-vis spectra and T_1_ relaxation time of Gd@Hb^Ce6-PEG^ nanoparticles before and after oxygenation. As a comparison, the oxygenation processes of pure Hb (4 mg/mL) were also carried out under the same conditions.

### Release of oxygen study

0.2 mL of oxy-Gd@Hb^Ce6-PEG^ (4 mg/mL) was quickly injected into 10 mL of de-oxygenated PBS and sealed. A dissolved oxygen detector (INESA Scientific Instrument Co., Ltd., China) was applied to record the real-time concentration of dissolved oxygen in the mixed solution, and an oxygen-dissociation curve was drawn 29. The process was repeated under the same conditions to draw the oxygen-dissociation curve of oxygenated Hb (oxy-Hb).

### Determination of singlet oxygen generation capacity of Gd@Hb^Ce6-PEG^ and oxy-Gd@Hb^Ce6-PEG^

200 μL of oxy-Gd@Hb^Ce6-PEG^ (7 mg/mL) was added to 3 mL of de-oxygenated PBS and sealed. Following the addition of oxygen sensor green (SOSG) (10 μmol) to the solution, the SOSG fluorescence emission spectra induced by oxy-Gd@Hb^Ce6-PEG^ was recorded before and after laser irradiation (0.6 mW/cm^2^) with an excitation wavelength of 488 nm, and a SOSG fluorescence intensity curve then drawn. The process was also performed under the same conditions to draw the SOSG fluorescence intensity change curve by Gd@Hb^Ce6-PEG^ (7 mg/mL).

### Cellular uptake

To investigate the cellular uptake of Gd@Hb^Ce6-PEG^, 4T1 breast cancer cells seeded in 6-well plates were incubated with Gd@Hb^Ce6-PEG^ nanoparticles at the concentration of 30 μg/mL for 1, 2 and 4 hrs, followed by washing twice with PBS and fixing with 4% paraformaldehyde solution. Fixed cells were then stained with 4′, 6-diamidino-2-phenylindole (DAPI) and observed with confocal laser scanning microscopy (SP5II, Leica) under 405 nm excitation.

### Cytotoxicity study

The mouse 4T1 tumor cell line was obtained from the Shanghai Institute of Cell Biology, Chinese Academy of Sciences (Shanghai, China). The cytotoxicity of Gd@Hb^Ce6-PEG^ was examined using cell counting kit-8 (CCK-8) assay. The 4T1 cells were seeded in 96-well microplates at a density of ~8000/well and incubated at 37 °C and 5% CO_2_. Then, the cells were incubated in fresh culture medium supplemented with 0 μg/mL, 3.75 μg/mL, 7.5 μg/mL, 15 μg/mL, 30 μg/mL, and 60 μg/mL of Gd@Hb^Ce6-PEG^ for 24 h. Subsequently, the cells were incubated in fresh culture medium containing 10 μL of CCK-8 reagent for another 2 h, followed by absorbance measurement at 450 nm with a multifunction microplate reader. The relative cell viability was calculated by comparing the absorbance intensity with the control cell samples.

### PDT on tumor cells

4T1 breast cancer cells at a density of ~8000/well in 96-well plates were incubated with Gd@Hb^Ce6-PEG^ or oxy-Gd@Hb^Ce6-PEG^ nanoparticles at the concentration of 30 μg/mL for 2 h, followed by 660 nm laser irradiation (12 mW/cm^2^) for 25 min and incubation for another 4 h in the dark. The cells without nanoparticles and subjected to laser irradiation were used as the control group. The relative cell viability was calculated by comparing the absorbance intensity with the control cell samples.

### Establishment of a tumor model

Experiments were conducted according to the protocols approved by the Institutional Animal Care and Use Committee of Tongji University. To construct the subcutaneous tumor model, 4T1 cells at a density of ~1×10^6^ dispersed in 100 μL of aseptic PBS were injected into the right foreleg or hindleg of BALB/C mice (22 g, 5 weeks, female, Shanghai Slac Laboratory Animal Co. Ltd.).

### *In vivo* MR imaging

The SE T_1_-weighted MR imaging of 4T1 tumor-bearing mice was conducted on the 3.0 T MRI scanner with a receiver coil for mice (Chenguang Med Tech, Shanghai, China). The mice were intravenously administrated with Gd@Hb^Ce6-PEG^ or Gd-DTPA in normal saline (Gd dose: 0.11 mmol/kg). Subsequently, the T_1_-weighted images of the liver, kidney, bladder, and tumor were acquired at six time points of 0.5 h, 1 h, 2 h, 4 h, 6 h, and 24 h after injecting Gd@Hb^Ce6-PEG^ or five time points; 2 min, 15 min, 1.5 h, 3 h, and 6 h after injecting Gd-DTPA. All images of kidneys and tumors except for background MR images were reconstructed with a maximum-intensity-projection (MIP) algorithm. Scanning parameters were set as follows: TR = 600 ms; TE = 20 ms; flip angle = 90°; FOV = 50×50 mm; voxel size = 0.2×0.198×1.0 mm; slice gap = 0.5 mm; slice thickness =1.0 mm; Total Scan Duration =4 min 44 s.

### *In vivo* tumor oxygenation evaluation

The level of oxygen in the tumor tissues was evaluated using the Visualsonic Vevo 2100 LAZER photoacoustic (PA) imaging system with the Oxy-hemo mode (750 nm and 850 nm) before and after the intravenous administration of Gd@Hb^Ce6-PEG^ or oxy-Gd@Hb^Ce6-PEG^ nanoparticles. The tumor-bearing mice with a tumor volume of approximately 80 mm^3^ were randomly divided into two groups (n = 3). The *in vivo* PA imaging of the tumor was recorded before and at 1 h, 2 h, and 4 h post-injection of plain Gd@Hb^Ce6-PEG^ or oxy-Gd@Hb^Ce6-PEG^ nanoparticles (Gd dose: 0.11 mmol/kg). The tumor oxygen saturation level of each mouse was determined by calculating the value of sO_2_ average total after placing a region of interest (ROI) of PA images covering the whole tumor.

To further evaluate the change in tumor hypoxia levels before and after the intravenous administration of Gd@Hb^Ce6-PEG^ or oxy-Gd@Hb^Ce6-PEG^, mice were sacrificed before and at 4 h and 24 h post-injection (Gd dose: 0.11 mmol/kg). The tumors were removed and fixed with tissue fixative and then dehydrated, embedded, and sliced for 4′,6-diamidino-2-phenylindole (DAPI) and hypoxia-inducible factor 1-alpha (HIF-1α) immunofluorescence staining. The tumor-bearing mice without the administration of Gd@Hb^Ce6-PEG^ served as controls.

### *In vivo* PDT and morphological MR imaging of tumor

The tumor-bearing mice were divided into 4 groups randomly (n = 4). Each group received different treatments as follows: (1) Saline injection; (2) Laser irradiation (1.0 W/cm^2^); (3) oxy-Gd@Hb^Ce6-PEG^ nanoparticles injection (Gd dose: 0.11 mmol/kg); (4) oxy-Gd@Hb^Ce6-PEG^ nanoparticles injection (Gd dose: 0.11 mmol/kg) plus laser irradiation (1.0 W/cm^2^). Subsequently, SE T_1_-weighted MR images of tumors were recorded for observing the size before treatment and at seven time points; 0.5 d, 1 d, 2 d, 4 d, 8 d, 12 d, and 16 d after the treatment*.* The body weight of each mouse was also recorded. The maximum height (a), maximum width (b), and maximum anteroposterior diameter (c) of the tumor were measured at the maximum transverse position of the MR and at the sagittal position. The volume of the tumor was calculated according to the formula: volume = a*b*c. The one-way analysis of variance (ANOVA) was used to compare the differences of mean relative tumor volume among groups. At the end of the experiment, the mice were sacrificed and the tumors were removed for further study.

### Diffusion-weighted imaging (DWI) for early evaluation of PDT efficacy of oxy-Gd@Hb^Ce6-PEG^

The multi-shot echo-planar imaging technique was conducted to acquire DW images. The scanning parameters were set as follows: TR = 1003 ms; TE = 95 ms; flip angle = 90°; FOV = 50×50 mm; voxel size = 0.78×0.93×2.0 mm; slice gap = 0.1 mm; slice thickness = 2.0 mm; b factors = 0, 1000 s/mm^2^; Total Scan Duration = 3 min 04 s. Apparent diffusion coefficient (ADC) maps were then constructed automatically and used for the measurement of ADC values.

### ADC measurement and data analysis

T_1_-weighted images were used for the localization of tumor lesions. The Philips MR Extended Workspace was used to process ADC maps. The ADC value in the tumor was calculated by drawing an ROI that covered the entire tumor. The one-way ANOVA was applied to compare the differences of the mean ADC values among groups.

### *In vivo* toxicity study

Blood toxicology study: Healthy mice (n=3) were intravenously administrated with Gd@Hb^Ce6-PEG^ (Gd dose: 0.11 mmol/kg) and the blood was collected on day 1 and day 14 post-injection, and routine serum biochemistry analysis was performed at the Shanghai Institute of Materia Medica. Saline-treated mice were used as the control group.

Organ toxicology study: Organ samples including lungs, heart, liver, spleen, kidneys, were removed, washed, fixed with tissue fixative, dehydrated, embedded, sliced (thickness: 4 microns), and stained with routine hematoxylin and eosin (H&E) using a standard protocol. The digital microscopy was used to observe the H&E staining images of organs. The saline-treated mice were used as the control group.

## Supplementary Material

Supplementary figures and tables.
