# Supplementary material for: Hemoglobin-mediated biomimetic synthesis of paramagnetic O2-evolving theranostic nanoprobes for MR imaging-guided enhanced photodynamic therapy of tumor
Source: Theranostics. 2020 Sep 19;10(25):11607–21. doi: 10.7150/thno.46228 (PMC7545996; doi:10.7150/thno.46228)
Supplement: Supplementary file 1 — Supplementary figures and tables. [file thnov10p11607s1.pdf]

Supporting Information

Hemoglobin-mediated biomimetic synthesis of paramagnetic O_2-evolving theranostic nanoprobes for MR imaging-guided enhanced photodynamic therapy of tumor

Xiudong Shi [1,2#], Weitao Yang [3#], Qiong Ma [1], Yang Lu [1], Yan Xu [3], Kexin Bian [3], Fengjun Liu [1,4], Chunzi Shi [1], Han Wang [2✉], Yuxin Shi [1,4✉], and Bingbo Zhang [3✉]

1. Department of Radiology, Shanghai Public Health Clinical Center, Fudan University, Shanghai 201508, China

2. Department of Radiology, Shanghai General Hospital, Shanghai Jiao Tong University School of Medicine, Shanghai 200080, China

3. The Institute for Translational Nanomedicine, Shanghai East Hospital; The Institute for Biomedical Engineering & Nano Science, Tongji University School of Medicine, Shanghai 200092, China

4. Translational Medicine Center, Shanghai Key Laboratory of Molecular Imaging, Shanghai University of Medicine and Health Sciences, Shanghai 201318, China

[#] These authors contributed equally to this study.

Corresponding Authors:

Bingbo Zhang, Email: bingbozhang@tongji.edu.cn

Yuxin Shi, Email: shiyx828288@163.com

Han Wang, Email: han.wang@shsmu.edu.cn

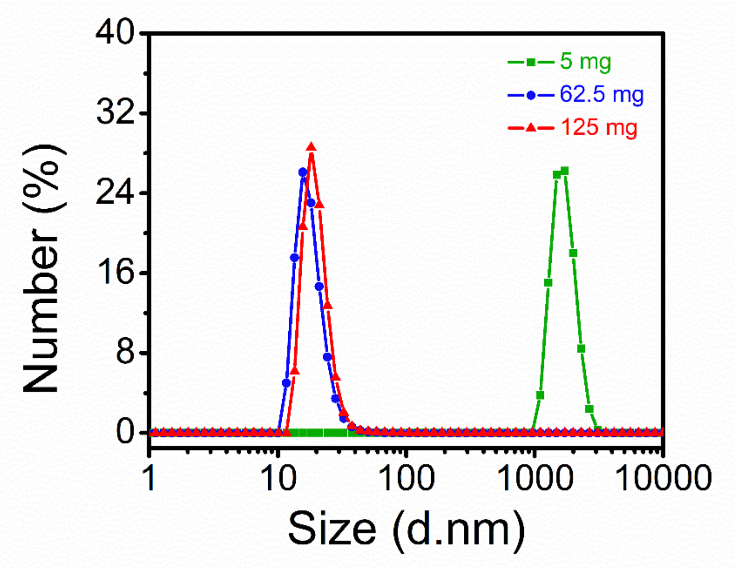

Figure S1. Hydrodynamic sizes of Gd@Hb$^{Ce6\text{-}PEG}$ nanoprobes synthesized with different concentrations of Hb (5 mg, 62.5 mg, and 125 mg).

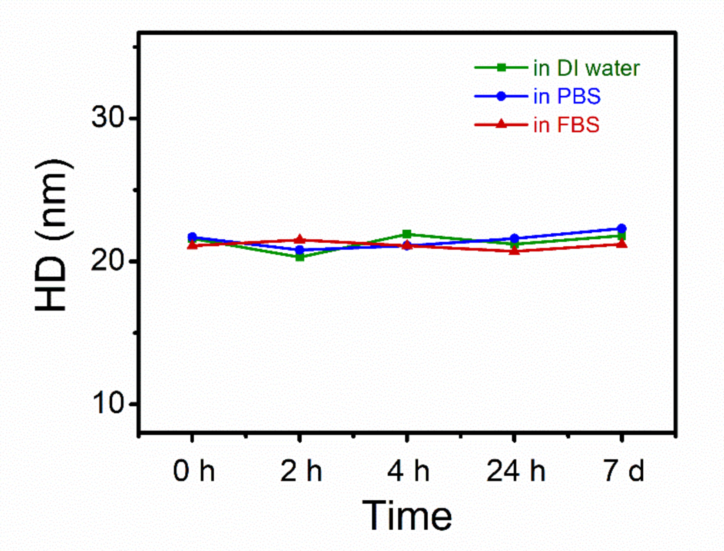

Figure S2. Hydrodynamic diameters (HDs) of Gd@Hb$^{Ce6\text{-}PEG}$ nanoparticles dispersed in deionized water (DI water), phosphate-buffered saline (PBS) or fatal bovine serum (FBS) *vs.* time.

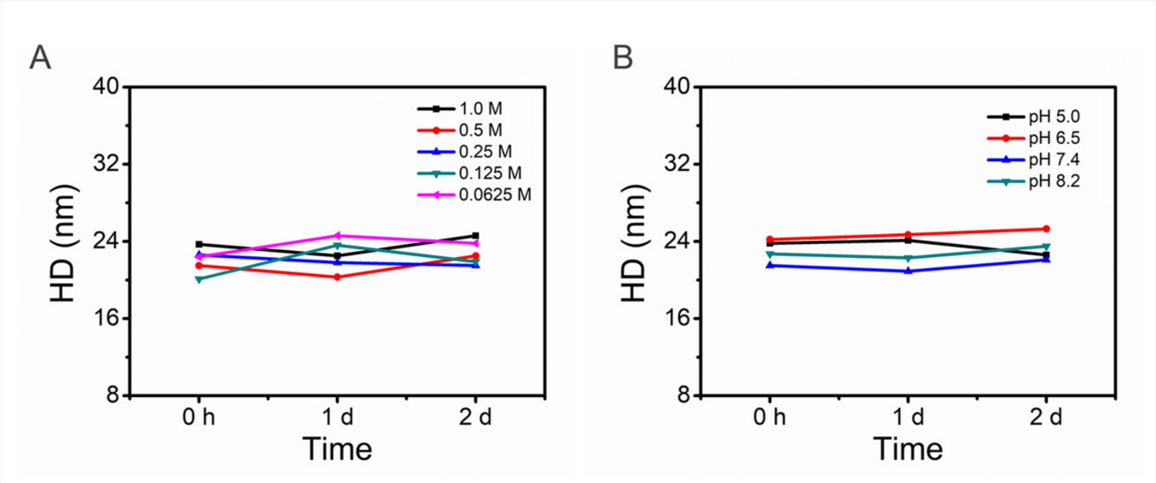

Figure S3. Hydrodynamic diameters (HDs) of Gd@Hb$^{Ce6\text{-}PEG}$ nanoparticles dispersed in sodium chloride water solutions with various ionic strengths (from 1.0 M to 0.0625 M) and buffer solutions with various pH values (pH varies from 5.0 to 8.2) vs. time.

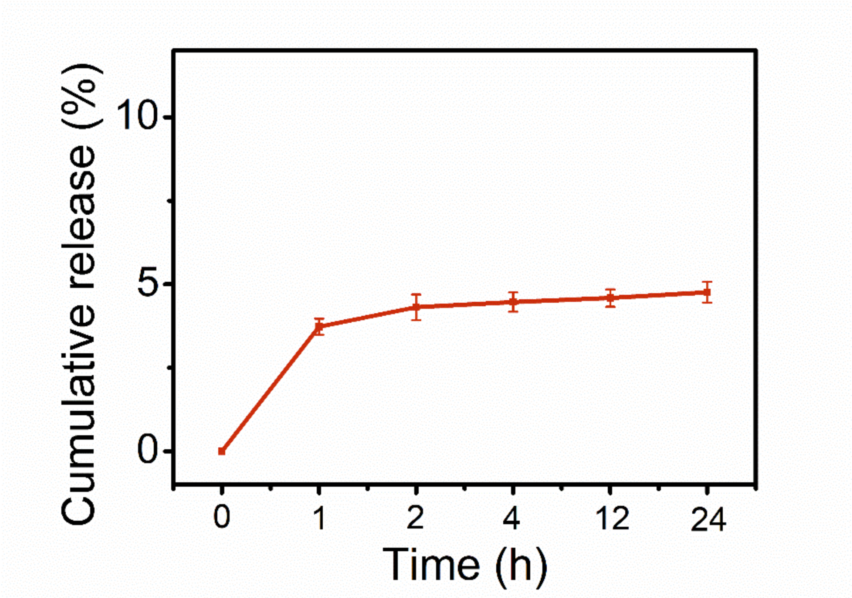

Figure S4. Ce6 release behavior of Gd@Hb$^{Ce6\text{-}PEG}$ nanoparticles.

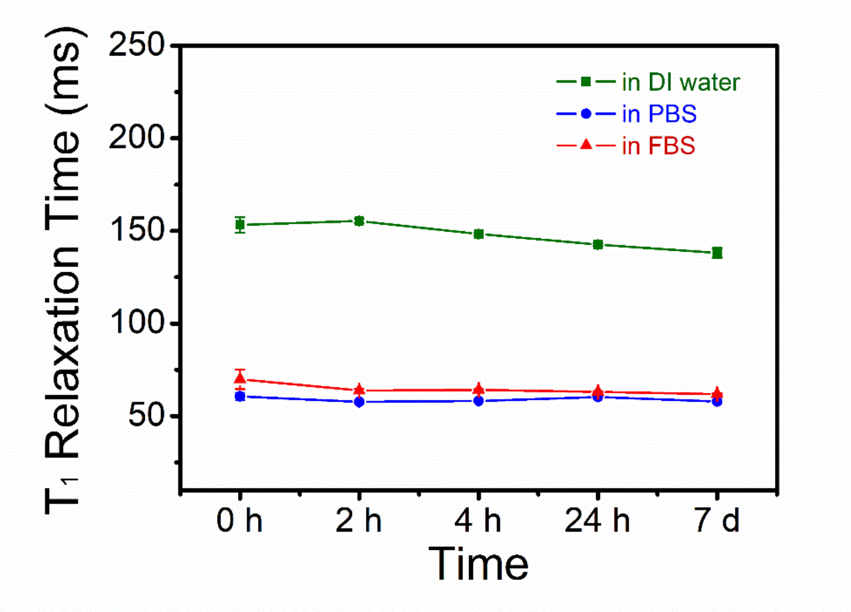

Figure S5. T_1 relaxation time of Gd@Hb$^{Ce6\text{-}PEG}$ nanoparticles dispersed in deionized water (DI water), PBS, or FBS *vs.* time.

Table S1. Gadolinium ion concentration measured by ICP-AES

Sample	Initial solution	Filtrate obtained at different times points	
		1 day	7 days
Gd concentration (ppm)	180.32	0.186	0.037
Leakage percentage (%)	/	<0.1 %	<0.02 %

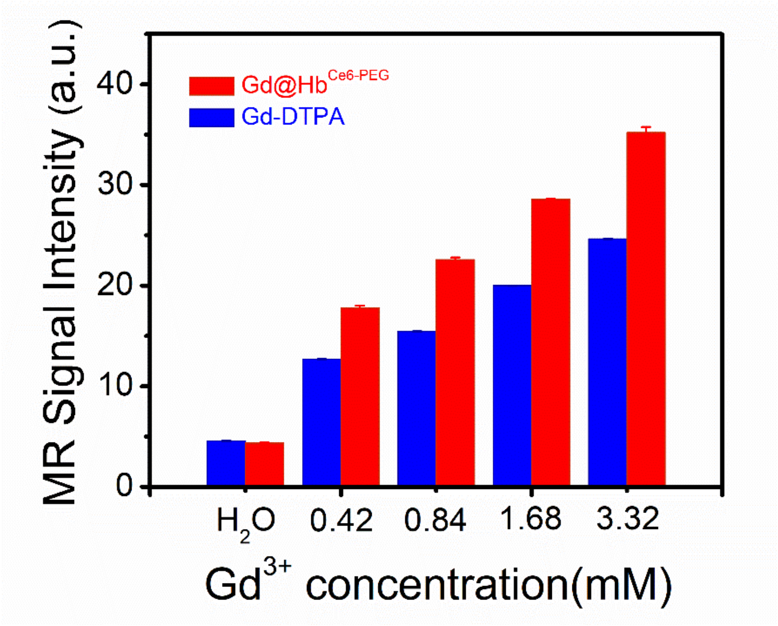

Figure S6. Quantified MR signal intensities of Gd@Hb$^{Ce6\text{-}PEG}$ and Gd-DTPA at different Gd^{3+} concentrations.

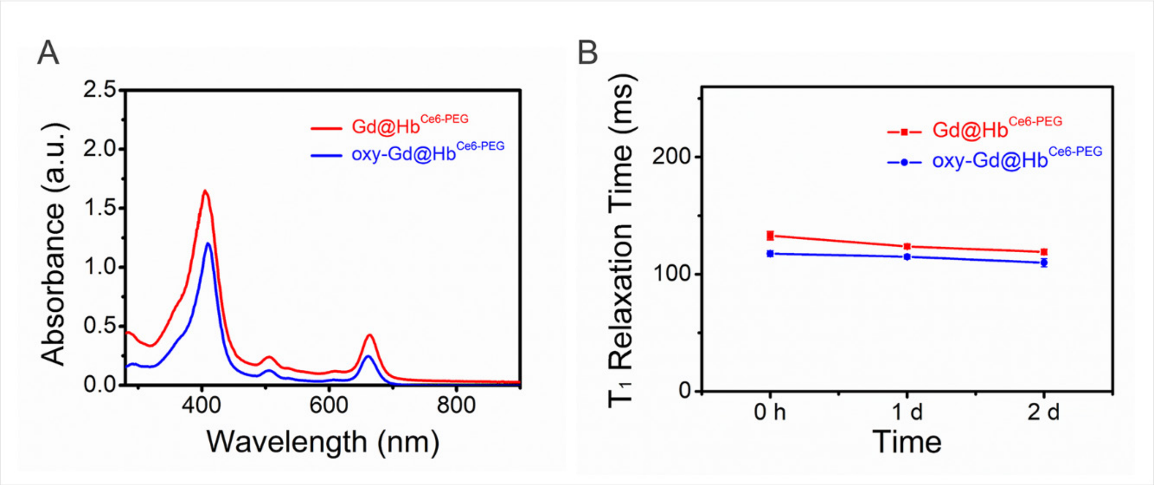

Figure S7. UV-vis spectra (A) and T_1 relaxation times (B) of Gd@Hb$^{Ce6\text{-}PEG}$ nanoparticles before and after oxygenation.

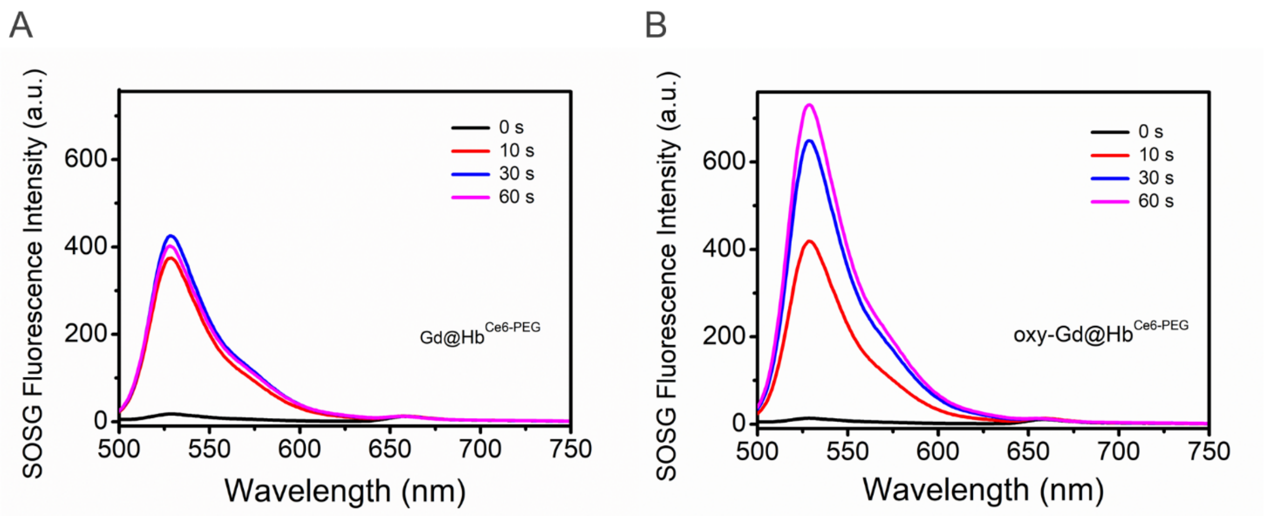

Figure S8. (A, B) Fluorescence emission spectra of (A) Gd@Hb$^{Ce6\text{-}PEG}$ and (B) oxy-Gd@Hb$^{Ce6\text{-}PEG}$ before and after irradiation at an excitation wavelength of 488 nm.

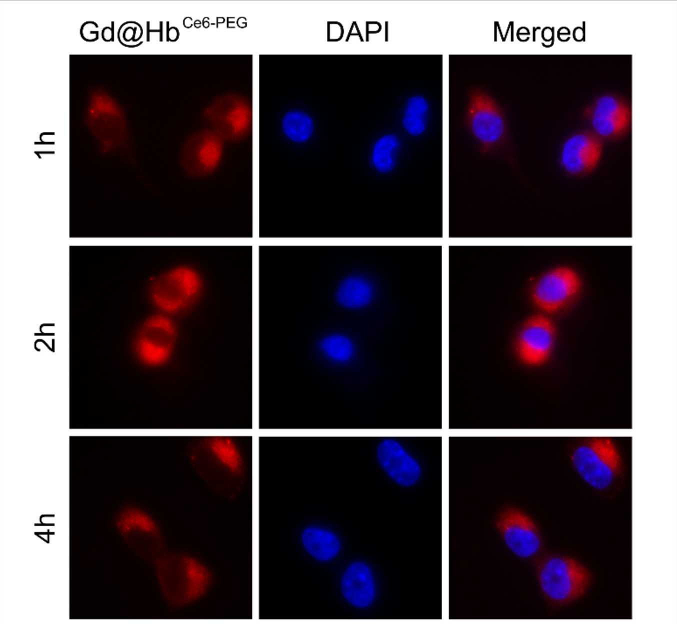

Figure S9. Cellular uptake of Gd@Hb$^{Ce6\text{-}PEG}$ nanoparticles. Confocal laser scanning microscopy images of 4T1 breast cancer cells after 1, 2, and 4 h of incubation with Gd@Hb$^{Ce6\text{-}PEG}$ (30 μg/mL) under 405 nm excitation (600 x). Blue: DAPI; red: Gd@Hb$^{Ce6\text{-}PEG}$.

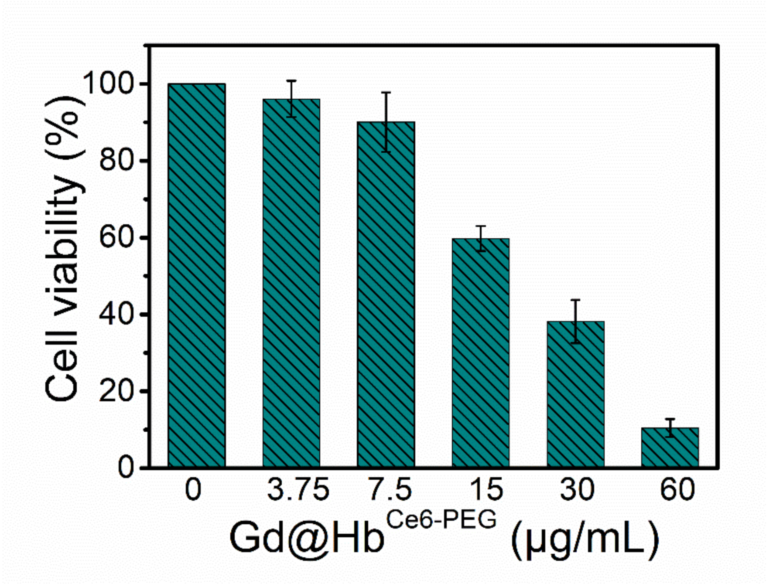

Figure S10. Viability of 4T1 cells incubated with Gd@Hb^Ce6-PEG plus the 660 nm laser irradiation at tested concentrations (0, 3.75, 7.5, 15, 30, and 60 μg/mL).

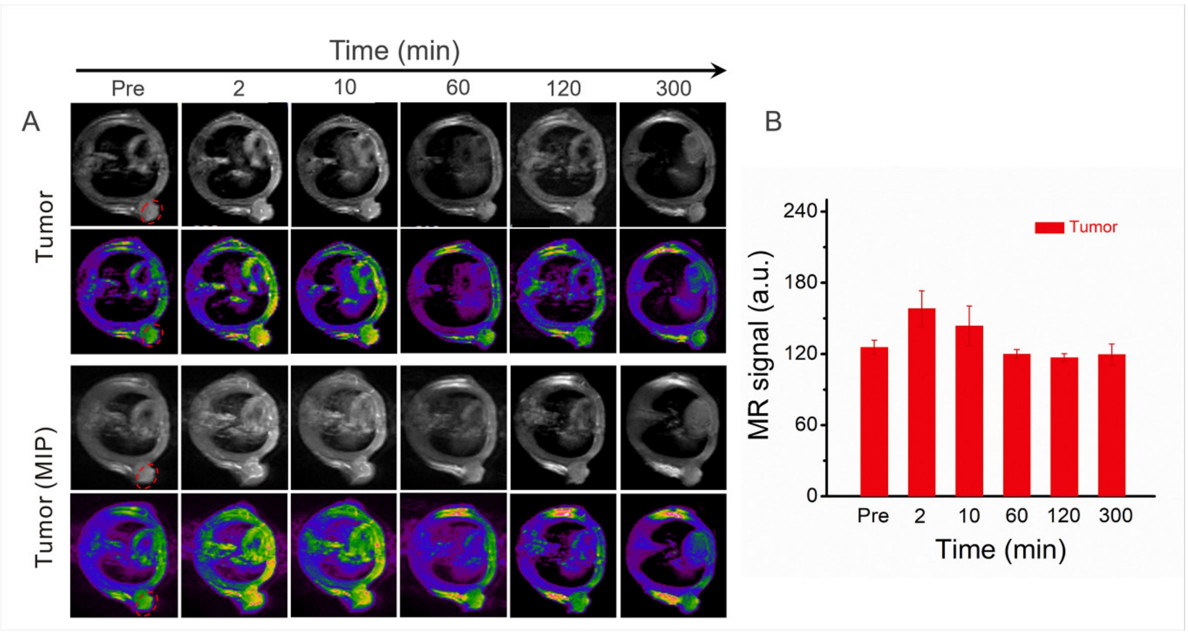

Figure S11. *In vivo* time-dependent MR imaging of the tumor in living mice with Gd-DTPA. (A) *In vivo* T_1-weighted MR images and the corresponding maximum intensity projection (MIP) images (red ellipse). (B) quantified MR signal intensity of 4T1 tumor with intravenous injection of Gd-DTPA (Gd dose: 0.11 mmol/kg).

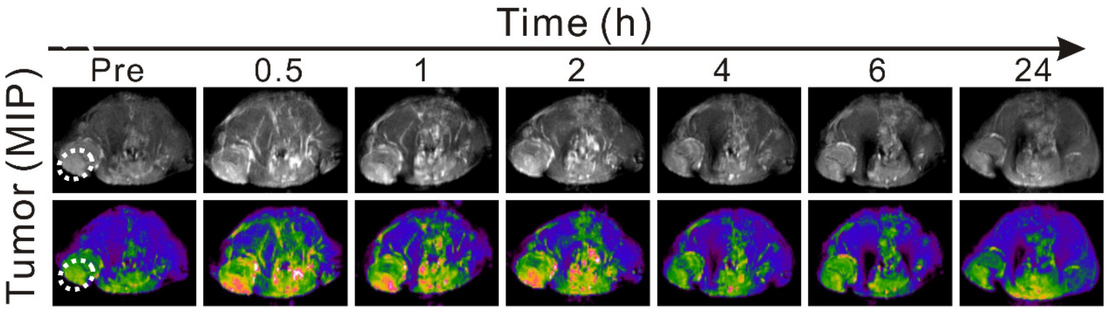

Figure S12. *In vivo* time-dependent MIP images of tumor with Gd@Hb$^{Ce6-PEG}$ (white ellipse).

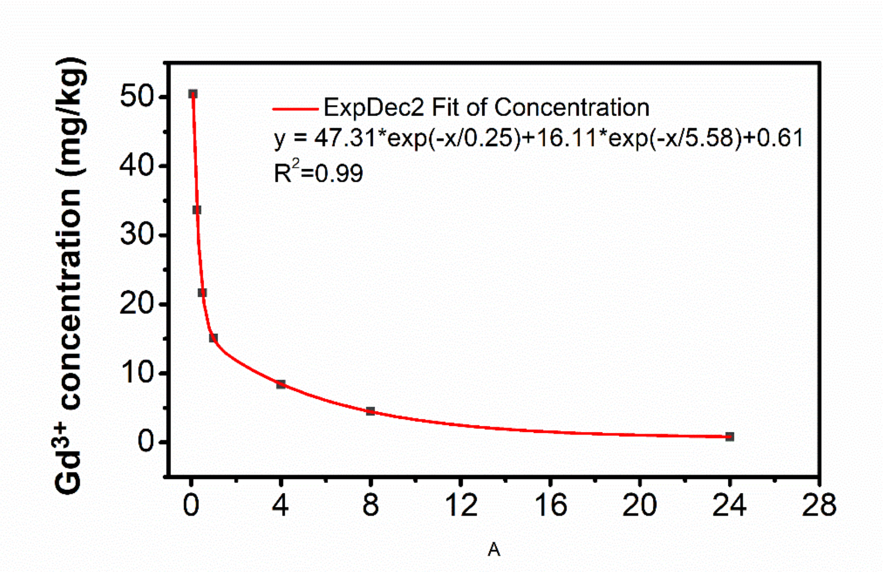

Figure S13. Measurement of circulation half-live of Gd@Hb$^{Ce6-PEG}$.

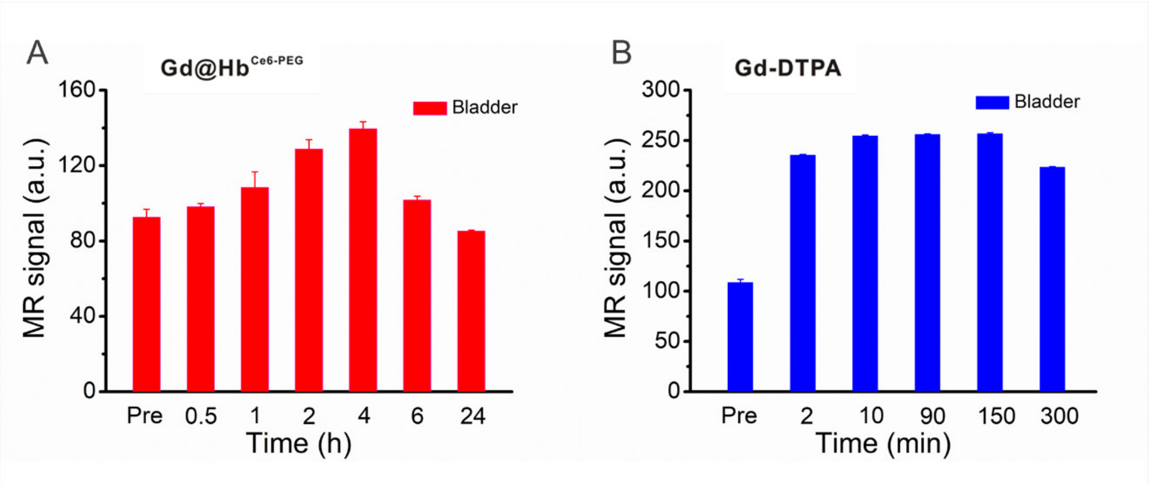

Figure S14. *In vivo* time-dependent quantified MR signal intensity of the bladder before and after intravenous injection of Gd@Hb$^{Ce6\text{-}PEG}$ (A) and Gd-DTPA (B).

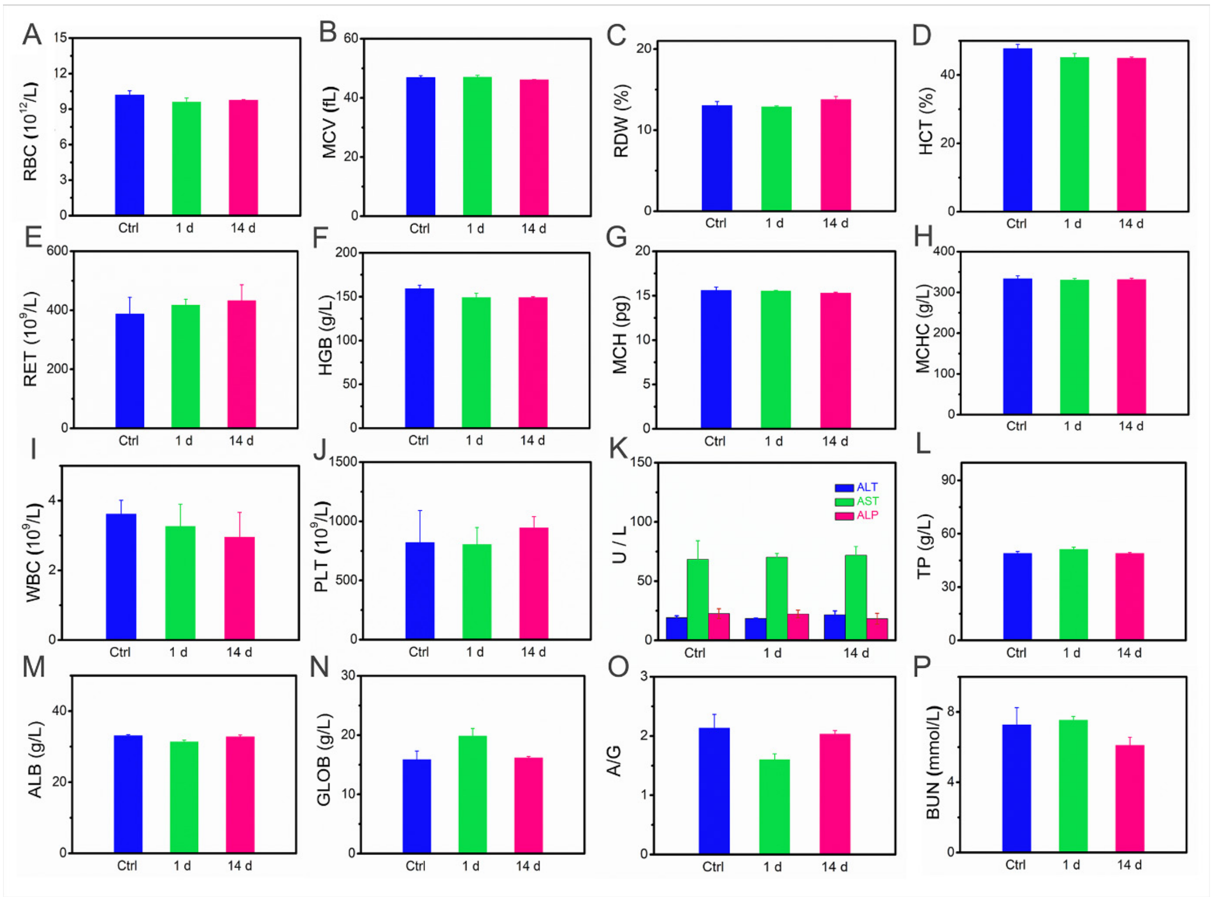

Figure S15. *In vivo* toxicity of Gd@Hb$^{Ce6\text{-}PEG}$ nanoparticles after intravenous injection. Routine blood tests with (A) red blood cells (RBC), (B) mean corpuscular volume (MCV), (C) red blood cell volume distribution width (RDW), (D) hematocrit (HCT), (E) reticulocyte (RET), (F) hemoglobin (HGB), (G) mean corpuscular hemoglobin (MCH), (H) mean corpuscular hemoglobin concentration (MCHC), (I) white blood cells (WBC), and (J) platelets (PLT). Biochemistry blood tests including (K) alanine aminotransferase (ALT), aspartate aminotransferase (AST), alkaline phosphatase (ALP), (L) total protein (TP), (M) albumin (ALB), (N) globulin (GLOB), (O) A/G (ALB/GLOB), and (P) blood urea nitrogen (BUN).

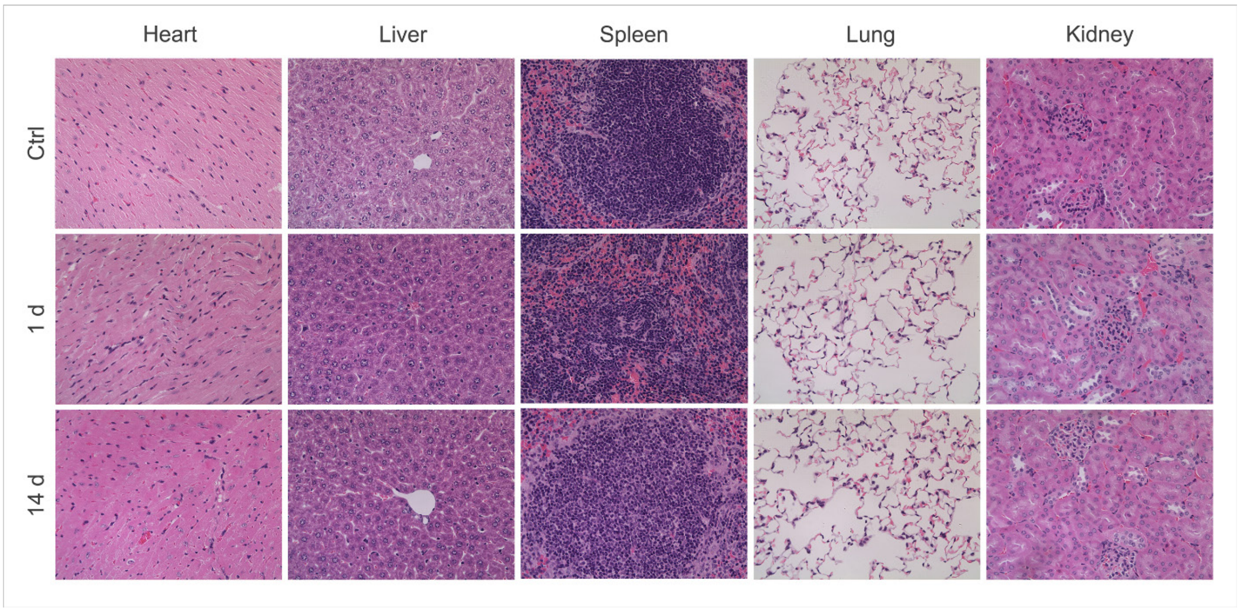

Figure S16. Representative H&E staining images of main organ tissues containing the heart, liver, spleen, lung, and kidney at day 1 and day 14 after intravenous injection (200 x). Saline-treated mice were set as a control group.
